# Proximal remote sensing: an essential tool for bridging the gap between high‐resolution ecosystem monitoring and global ecology

**DOI:** 10.1111/nph.20405

**Published:** 2025-01-23

**Authors:** Zoe Amie Pierrat, Troy S. Magney, Will P. Richardson, Benjamin R. K. Runkle, Jen L. Diehl, Xi Yang, William Woodgate, William K. Smith, Miriam R. Johnston, Yohanes R. S. Ginting, Gerbrand Koren, Loren P. Albert, Christopher L. Kibler, Bryn E. Morgan, Mallory Barnes, Adriana Uscanga, Charles Devine, Mostafa Javadian, Karem Meza, Tommaso Julitta, Giulia Tagliabue, Matthew P. Dannenberg, Michal Antala, Christopher Y. S. Wong, Andre L. D. Santos, Koen Hufkens, Julia K. Marrs, Atticus E. L. Stovall, Yujie Liu, Joshua B. Fisher, John A. Gamon, Kerry Cawse‐Nicholson

**Affiliations:** ^1^ Jet Propulsion Laboratory California Institute of Technology Pasadena CA 91011 USA; ^2^ Department of Plant Sciences University of California Davis CA 95616 USA; ^3^ Biological & Agricultural Engineering University of Arkansas Fayetteville AK 72701 USA; ^4^ Center for Ecosystem Science and Society Northern Arizona University Flagstaff AZ 86011 USA; ^5^ School of Informatics, Computing & Cyber Systems Northern Arizona University Flagstaff AZ 86011 USA; ^6^ Department of Environmental Sciences University of Virginia Charlottesville VA 22904 USA; ^7^ School of the Environment The University of Queensland Brisbane 4072 Qld Australia; ^8^ CSIRO, Space and Astronomy Kensington 6151 WA Australia; ^9^ School of Natural Resources and the Environment University of Arizona Tucson AZ 85721 USA; ^10^ Department of Geographical and Sustainability Sciences University of Iowa Iowa City IA 52242 USA; ^11^ Climate Monitoring Group, Department of Meteorology, Institute of Geosciences University of Bonn 53121 Bonn Germany; ^12^ Copernicus Institute of Sustainable Development Utrecht University 3584 Utrecht the Netherlands; ^13^ Forest Ecosystems & Society, Oregon State University 321 Richardson Hall Corvallis OR 97331 USA; ^14^ Department of Geography University of California Santa Barbara CA 93106 USA; ^15^ O'Neill School of Public and Environmental Affairs Indiana University Indiana 47405 USA; ^16^ Department of Geography, Environment, and Spatial Sciences Michigan State University East Lansing MI 48824 USA; ^17^ Department of Civil and Environmental Engineering Utah State University Logan UT 84322 USA; ^18^ JB Hyperspectral Devices 40225 Düsseldorf Germany; ^19^ University of Milano – Bicocca 20126 Milano Italy; ^20^ Laboratory of Bioclimatology, Department of Ecology and Environmental Protection Poznan University of Life Sciences 60‐637 Poznan Poland; ^21^ Forestry and Environmental Management University of New Brunswick Fredericton NB E3B 5A3 Canada; ^22^ Climate & Ecosystem Sciences Division Lawrence Berkeley National Laboratory Berkeley CA 94702 USA; ^23^ Institute of Geography University of Bern 3012 Bern Switzerland; ^24^ Oeschger Centre for Climate Change Research University of Bern 3012 Bern Switzerland; ^25^ National Institute of Standards and Technology 100 Bureau Dr. Gaithersburg MD 20899 USA; ^26^ NASA Goddard Space Flight Center 8800 Greenbelt Rd Greenbelt MD 20771 USA; ^27^ Schmid College of Science and Technology Chapman University 1 University Dr. Orange CA 92866 USA; ^28^ CALMIT, School of Natural Resources University of Nebraska – Lincoln Lincoln NE 68588 USA

**Keywords:** biodiversity, canopy structure, ecosystem flux, eddy covariance, phenology, proximal remote sensing, scaling, spectral biology

## Abstract

A new proliferation of optical instruments that can be attached to towers over or within ecosystems, or ‘proximal’ remote sensing, enables a comprehensive characterization of terrestrial ecosystem structure, function, and fluxes of energy, water, and carbon. Proximal remote sensing can bridge the gap between individual plants, site‐level eddy‐covariance fluxes, and airborne and spaceborne remote sensing by providing continuous data at a high‐spatiotemporal resolution. Here, we review recent advances in proximal remote sensing for improving our mechanistic understanding of plant and ecosystem processes, model development, and validation of current and upcoming satellite missions. We provide current best practices for data availability and metadata for proximal remote sensing: spectral reflectance, solar‐induced fluorescence, thermal infrared radiation, microwave backscatter, and LiDAR. Our paper outlines the steps necessary for making these data streams more widespread, accessible, interoperable, and information‐rich, enabling us to address key ecological questions unanswerable from space‐based observations alone and, ultimately, to demonstrate the feasibility of these technologies to address critical questions in local and global ecology.

**Table:** 

	Contents	
	Summary	420
I.	Introduction: why proximal remote sensing?	420
II.	Ecological applications of proximal remote sensing	421
III.	Synergies	426
IV.	The case for a network	427
V.	A path forward for networking proximal remote sensing data	428
VI.	Conclusions	429
	Acknowledgements	429
	References	430

## I. Introduction: why proximal remote sensing?

Our ability to anticipate and plan for future changes to the climate system depends on a mechanistic understanding of water, energy, and carbon fluxes in terrestrial ecosystems. Global understanding of these fluxes is made possible by scaling up insights from local or site‐level research to answer the grand challenges in global ecology (Schimel *et al*., 2019): what is the primary productivity of the globe and how is it controlled?; how much carbon does the biosphere store and how could it change?; how does direct human exploitation of the biosphere affect productivity and carbon storage?; what is the biological diversity of the world and how does it affect the function and stability of ecosystems?

Site‐level research, primarily from towers using the eddy‐covariance technique, has enabled considerable insight into the past, present, and future status of ecosystem fluxes and their environmental sensitivities (Baldocchi, 2020; Baldocchi *et al*., 2024). However, eddy‐covariance measurements are limited in their spatial extent, over‐sample some biomes (e.g. temperate forests and agriculture) and under‐sample others (e.g. drylands, tropical forests, and boreal forests), are often short‐lived (with relatively few sites providing multiple decades of measurements), provide area‐averaged estimates across the tower footprint, are restricted to flat terrain, and are subject to gaps and uncertainties associated with data processing (Hollinger & Richardson, 2005; Mauder *et al*., 2013; Chu *et al*., 2021; Villarreal & Vargas, 2021). Remote sensing offers a means for upscaling and gap‐filling eddy‐covariance data, and for quantifying and monitoring biological processes that drive observed fluxes. To appropriately scale remote sensing data to the satellite and address major questions in global ecology, site‐level data are needed.

Although space‐based observations hold great potential for understanding ecosystems (Stavros *et al*., 2017; Schimel *et al*., 2019), the ecosystem dynamics controlling global change biology often occur at spatiotemporal scales that are not well captured by satellites due to their inherently limited spatiotemporal resolution (Jantol *et al*., 2023). Recent work has shown a rapid decrease in information content from multi‐spectral and thermal imagery going from a 1‐ to 5‐d revisit time, with thermal data losing up to *c*. 80% of information content at a 6‐d revisit (Cawse‐Nicholson *et al*., 2022). Even with a 1‐d revisit time, many of the aforementioned ecosystem processes happen at a sub‐daily timescale, and information gain is projected to increase with sub‐daily data acquisition (Cawse‐Nicholson *et al*., 2021, 2023). Furthermore, the spatial averaging that occurs with space‐based observations obscures heterogeneous ecosystem components of observed fluxes and processes. Aircraft‐ and UAV‐based assessment of ecosystem dynamics allows a more detailed spatial view than traditional satellites (Maguire *et al*., 2021; Berger *et al*., 2022) and has shown utility for algorithmic development particularly when paired with coordinated field campaigns (Chadwick *et al*., 2020). However, aircraft remote sensing can be costly, and infrequent overpasses can undermine its ability to resolve temporal uncertainties in space‐based remote sensing. Temporal uncertainties may be partially overcome with repeat UAV‐based assessment, which has undergone significant advancements in the past several years, although this type of assessment is highly time‐intensive. Thus, in order for remote sensing to resolve these grand challenges in ecology (Schimel *et al*., 2019) we must resolve spatiotemporal gaps, quantify errors and uncertainties, and develop new algorithms which draw mechanistic ties between observed remote sensing signals and biologic processes (Pierrat *et al*., 2024a).

Tower‐mounted remote sensing (hereby referred to as proximal remote sensing) provides relatively low‐cost, high spatial, temporal, and spectral resolution measurements that enhance the capabilities of space‐ and airborne sensors at the site level, enabling a more direct link to co‐located eddy‐covariance measurements (Gamon, 2015). Proximal remote sensing can be used to develop new remote sensing methods, validate satellite measurements and products (Parazoo *et al*., 2019), drive and test the representation of ecosystem processes in models (Raczka *et al*., 2019), reveal spatial heterogeneity in fluxes within individual eddy‐covariance tower footprints (Chu *et al*., 2021) and scale site‐level measurements to the landscape seen by spaceborne instruments (Farella *et al*., 2022) – providing unprecedented insights into the physical, biological, and species‐specific processes that drive ecosystem fluxes.

**Fig. 1:**
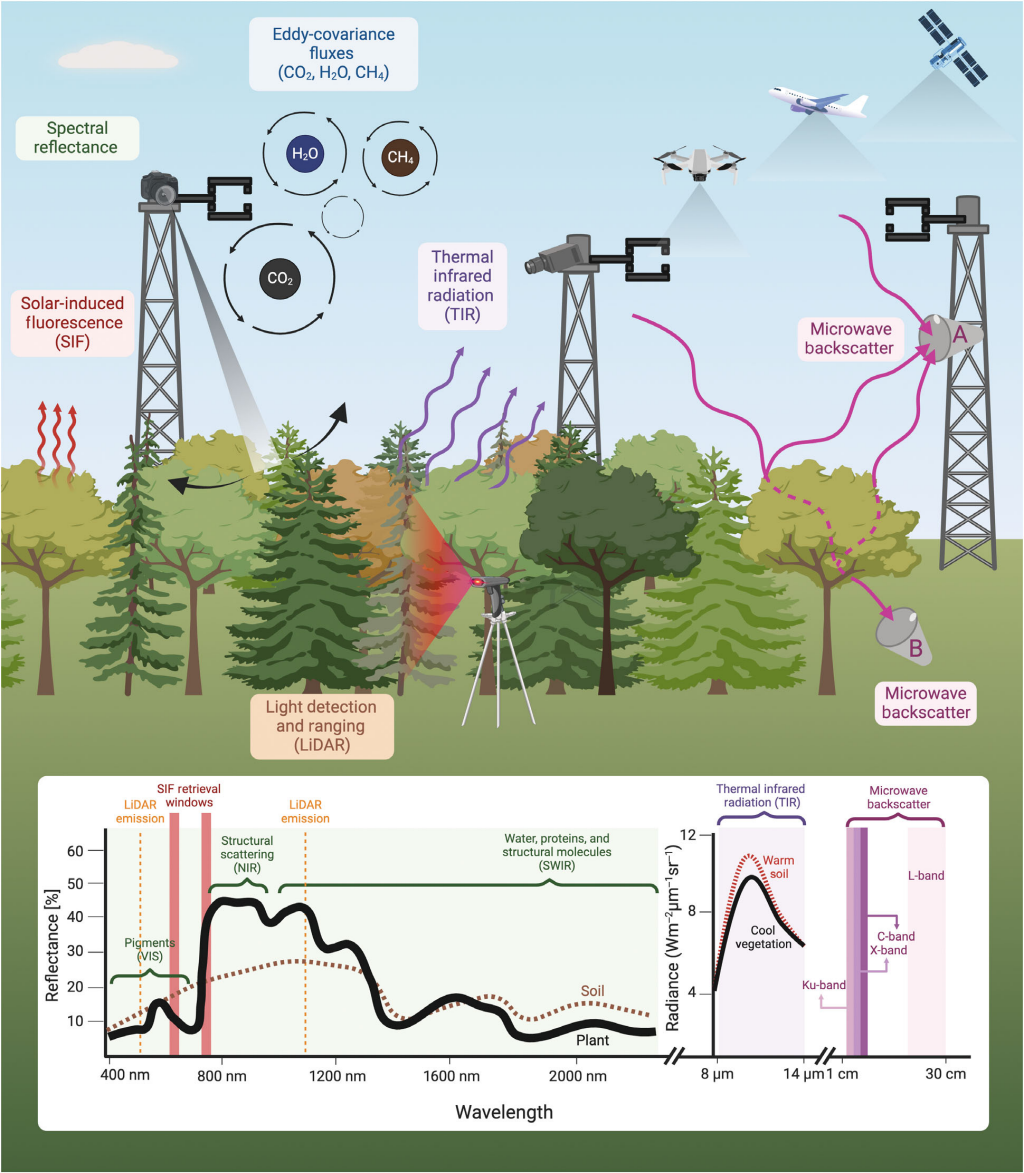
Overview of proximal remote sensing instruments at a flux tower site. Shown are three eddy‐covariance towers with sonic anemometers collecting data to derive ecosystem fluxes. Shown for spectral reflectance and solar‐induced fluorescence (SIF) is a hyperspectral sensor with a narrow field‐of‐view (FOV) and multi‐directional scanning capabilities (Sections II.1 and II.2). We also show the direct emission of SIF from the forest canopy (Section II.2). For thermal infrared radiation, we show a fixed thermal camera and thermal radiation coming from the canopy (Section II.3). For microwave, we show two potential arrangements with antenna A receiving direct signals from under open‐sky conditions as well as signals that are reflected from the underlying vegetated surface, and antenna B receiving a direct signal that is propagated downward through the vegetation canopy and attenuated by its moisture content (Section II.4). We also show a light detection and ranging (LiDAR) instrument emitting light to get a 3D representation of canopy structure (Section II.5). Above the forest are a drone, aircraft, and satellite to emphasize the potential of proximal remote sensing to complement observations across scales. In the inset plot, we show sample reflectance spectra for vegetation and wet soil and highlight key wavelength ranges for spectral reflectance. We also show typical SIF retrieval windows and LiDAR emission windows. Next to the reflectance spectra, we show sample radiance in the thermal infrared region, with example spectra for warm soil and cool vegetation. Finally, we show key measurement wavelength bands for microwave backscatter. Created in BioRender. Pierrat, Z. (2025) https://BioRender.com/z36d893.

Here, we review five different types of proximal remote sensing that cover ecosystem structure, composition, and function: visible‐to‐shortwave infrared (VSWIR) spectral reflectance, solar‐induced chlorophyll (Chl) fluorescence (SIF), longwave thermal infrared radiation (TIR), microwave backscatter, and light detection and ranging (LiDAR) (Fig. 1; Section II). Alone or synergistically (Section III), these data have the potential to resolve three key questions necessary to advance global ecology:

1. What is the scale dependence (spectral, spatial, and temporal) of ecological processes and fluxes?
2. What are the underlying physical and biological drivers of observed remote sensing signals?
3. How can new technologies, synergies, algorithms, and models developed at the site advance our understanding of global ecology at scale?

Answering these questions hinges on the community's ability to develop proximal remote sensing networks that will support ecosystem and flux science, existing and upcoming satellite missions, and model development and validation (Section IV). Despite the importance and utility of proximal remote sensing data, there remains a significant barrier to entry for researchers to collect and exploit these data and thus an urgent need for democratization and standardization of proximal remote sensing products (Section V). While attempts have been made to coordinate proximal remote sensing data streams (Gamon *et al*., 2006, 2010; Balzarolo *et al*., 2011; Rasaiah *et al*., 2013; Gamon, 2015), these efforts have been encumbered by a lack of consistent funding, an absence of suitable data standardization and archiving protocols, insufficient incentive for individual participation, and have remained limited in their locational extent (primarily individual sites in the United States, Europe, and Australia). With new and upcoming satellite missions enabling multi‐sensor studies and further insight into plant function (Stavros *et al*., 2017; Schimel *et al*., 2019), the time is right to enable community‐led initiatives toward these goals and to align these initiatives with intergovernmental and commercial aircraft and satellite programs. The eddy covariance and remote sensing communities can leverage momentum from the AmeriFlux Year of Remote Sensing initiative, including lessons learned from the international FLUXNET network coordination (Pastorello *et al*., 2020), and the broader push toward open and equitable science to develop such a network (Ramachandran *et al*., 2021). To this end, we provide interested readers with practical recommendations for data collection, including best practices, technical considerations, and sample metadata (Supporting Information Notes S1, S2) for different instrument types (Tables S1–S3) and to facilitate data access, we have compiled resources where readers can access existing data streams (Notes S3; Tables S4, S5).

## II. Ecological applications of proximal remote sensing

### 1. Ecosystem structure and function with visible‐to‐shortwave infrared spectral reflectance

Spectral reflectance remote sensing is the passive detection of reflected solar radiation in the VSWIR (*c*. 400–2500 nm) range (common instruments in Table S1). Spectral reflectance is most commonly linked with eddy‐covariance‐based gross primary productivity (GPP) using the light use efficiency (LUE) model (Monteith, 1972; Gamon, 2015). Under this model, GPP is defined as:

(Eqn 1) $$\mathrm{GPP}=\mathrm{PAR}\times {f\mathrm{PAR}}_{\mathrm{chl}}\times {\mathrm{LUE}}_{P}$$

where PAR is the photosynthetically active radiation, *f*PAR_chl_ is the fraction of that light absorbed by Chl, and LUE_P_ is the LUE of photosynthesis (i.e. the fraction of the absorbed light which is used for photosynthesis). Based on this model, remote sensing metrics that are sensitive to *f*PAR_chl_ (which is a function of canopy structure and Chl content) or LUE_P_ (which is a function of the regulation of light energy by plants, typically modulated by pigment concentrations) are particularly useful. Metrics to approximate *f*PAR_chl_ and LUE_P_ have been developed based on the dynamic shape of plant spectral reflectance (Fig. 1) and its sensitivity to changes in plant pigment concentrations. Traditionally, wavelength regions have been combined using two or more spectral channels to produce a vegetation index (reviewed in Inoue *et al*., 2008; Zeng *et al*., 2022) although more complex techniques are emerging. Importantly, all these parameters (PAR, *f*PAR_chl_, and LUE_P_) are highly temporally dynamic. Thus, spectral responses to changes in pigment, water, or other biochemical concentrations are often developed at a proximal level to draw a more mechanistic connection between changes in plant traits, states, and functions and their spectral response. This type of proximal sensing has the additional benefit of helping us understand the spatiotemporal scale dependence of these processes.

Reflectance in the near‐infrared (NIR) region is primarily sensitive to plant biophysical properties, including leaf thickness, density, leaf angle distribution, and mesophyll structure (Fig. 1) – enabling remote sensing inferences into canopy structure, leaf area index (LAI), and biomass, all of which modulate *f*PAR_chl_ (Tucker, 1979; Slaton *et al*., 2001; Myneni *et al*., 2002; Yang *et al*., 2023). The red‐edge transition region between VIS and NIR (*c*. 700–750 nm) is also sensitive to subtle changes in Chl concentrations (Gitelson *et al*., 2002, 2003, 2005), which additionally modulates *f*PAR_chl_ and can be linked to canopy nitrogen (Magney *et al*., 2017). NIR‐based metrics tend to track GPP well over the seasonal cycle at sites where LAI, biomass, and Chl content tend to co‐vary with GPP, such as annual well‐watered crops, deciduous forests, and grasslands (Baldocchi *et al*., 2020; Dechant *et al*., 2020; Lin *et al*., 2022). These observations are consistent from proximal to spaceborne scales (Badgley *et al*., 2019; Baldocchi *et al*., 2020), as these structural traits are expected to vary more slowly through time, but can be heavily influenced by viewing and solar geometries throughout the day – which can be thoroughly investigated at the site scale with proximal remote sensing (Hilker *et al*., 2008).

In ecosystems where changes in physiology are decoupled from changes in structure, such as evergreen forests (Magney *et al*., 2019; Z. Pierrat *et al*., 2022) and dry mixed grasslands and shrublands (Wang *et al*., 2022), NIR‐based reflectance is typically insufficient for capturing rapid changes in plant function. Monitoring changes in the VIS region with proximal remote sensing therefore enables estimation of plant pigments and biochemical changes related to plant regulation of light energy (LUE_P_), which can change dramatically from sub‐daily to seasonal time scales (Garbulsky *et al*., 2011; Cheng *et al*., 2020; Seyednasrollah *et al*., 2020). A prime example is the Photochemical Reflectance Index (PRI), which is sensitive to xanthophyll pigment conversion states, which regulate plant photoprotection and thus modulate LUE_P_ (Gamon *et al*., 1992, 1997). While PRI has been shown to be effective for tracking short‐term (diurnal) changes in LUE_P_, it is confounded by longer‐term (constitutive) changes in pigments (Garbulsky *et al*., 2011; Wong & Gamon, 2015). To account for longer‐term changes in carotenoid pigments, the Chl: Carotenoid Index (CCI) has shown promise at proximal (Wong *et al*., 2020; Z. A. Pierrat *et al*., 2022, 2024b) and satellite (Gamon *et al*., 2016) scales.

Plant water status can also change over the course of a day and be estimated using reflectance in the longer wavelengths of the NIR and SWIR regions, which are characterized by water absorption features (*c*. 970, 1400 and 1900 nm) (Gao & Goetz, 1994; Sims & Gamon, 2003). Additionally, empirical estimates of plant lignin, cellulose, proteins, nutrients, and phenolics have been achieved in the SWIR region (Curran, 1989; Ceccato *et al*., 2001; Kokaly & Skidmore, 2015), but limited research has been done linking tower‐based SWIR to canopy processes.

Recent work has developed new approaches to predict plant traits, states, and functions, such as photosynthesis (Dechant *et al*., 2017; Meacham‐Hensold *et al*., 2020), pigments (Cheng *et al*., 2020), stomatal conductance (Wong, 2023), disease (Zarco‐Tejada *et al*., 2018; Gold *et al*., 2020), leaf nitrogen, carbon, calcium, sulfur, phosphorus, sugars, starches, leaf mass per area, and leaf water content (Ely *et al*., 2019; Z. Wang *et al*., 2020; Burnett *et al*., 2021; Féret *et al*., 2021; Verrelst *et al*., 2021; Tagliabue *et al*., 2022). These new approaches use the entire spectrum and physically based (i.e. inversion of radiative transfer models (Pacheco‐Labrador *et al*., 2019)), machine learning, and statistical (e.g. principal component analysis, partial least squares regression, neural networks, and random forests) or hybrid approaches. These new methods may be scaled to space‐based applications, but proximal data are needed to help disentangle noise due to background influence, mixed pixels, and limitations in temporal resolution.

Beyond plant structure and function, hyperspectral reflectance data can identify species and biodiversity, which can be helpful for understanding species composition at sub‐satellite pixel scales (Ballanti *et al*., 2016; Wang & Gamon, 2019; Cavender‐Bares *et al*., 2020; Gholizadeh *et al*., 2022; Kamoske *et al*., 2022). Notably, biodiversity is highly scale‐dependent (Cavender‐Bares *et al*., 2020; Gonzalez *et al*., 2020; Gamon, 2023). Thus, research focused on scaling remotely sensed biodiversity measures will considerably advance our ability to track biodiversity in space and time, and is enabled by high‐resolution proximal remote sensing data (Ustin & Gamon, 2010; Chase *et al*., 2018). Understanding biodiversity and species composition within a flux tower footprint enables both a better understanding of contributions to the flux signal, as well as the relationship between system functional diversity and productivity (Gamon, 2023). Due to the high‐spatiotemporal resolution of these processes, these understandings would not be possible with spaceborne remote sensing alone and can help interpret spaceborne observations.

### 2. Carbon fluxes and plant health with Solar‐induced fluorescence

Solar‐induced Chl fluorescence is a weak light signal emitted in the red and NIR (650–850 nm with two peaks at 687 nm and 740 nm) during the light reactions of photosynthesis when excited Chl molecules return to their ground state. Because of the direct link to leaf physiology, SIF is typically used as a proxy for eddy‐covariance‐derived GPP (Frankenberg & Berry, 2017; Porcar‐Castell *et al*., 2021) and can be expressed similarly to the LUE model of photosynthesis. Canopy level SIF is commonly expressed as:

(Eqn 2) $$\mathrm{SIF}=\mathrm{PAR}\times {f\mathrm{PAR}}_{\mathrm{chl}}\times \phi_{F}\times f_{\mathrm{esc}}$$

where PAR is the photosynthetically active radiation, *f*PAR_chl_ is the fraction of light absorbed by Chl, φ_F_ is the yield of fluorescence (i.e. the fraction of the absorbed light which is re‐emitted as fluorescence), and *f*_esc_ is the fraction of emitted SIF photons that escape the canopy to be detected by a sensor. These drivers all have spatiotemporal scale dependencies that can go unresolved with spaceborne remote sensing.

While satellite remote sensing of SIF has revealed strong correlations between SIF and GPP at eddy‐covariance sites across the globe (Guanter *et al*., 2014; Sun *et al*., 2017), proximal SIF data have illuminated nuance in the SIF–GPP relationship and shed light on the mechanisms influencing their relationship (current available data in Table S4). SIF and GPP share the common drivers of PAR and *f*PAR_chl_, which can explain part of the strong covariation between SIF and GPP at spaceborne scales or in ecosystems where canopy structure and productivity are tightly coupled, such as crops (Dechant *et al*., 2020). Depending on the spatiotemporal scale, $\phi_{F}$ and the yield of photosynthesis ($\phi_{P}$, leaf‐level LUE_P_) may not always co‐vary, leading to divergence in the SIF‐GPP relationship (Magney *et al*., 2020; Pierrat *et al*., 2024b). In particular, tower‐scale SIF has identified and mechanistically explained divergence during periods of heat stress (Wieneke *et al*., 2018; Wohlfahrt *et al*., 2018; Martini *et al*., 2022), cold stress (Z. Pierrat *et al*., 2022), drought or water stress (Buddenbaum *et al*., 2015; Butterfield *et al*., 2023), high VPD (Paul‐Limoges *et al*., 2018), high light (Miao *et al*., 2018; Kim *et al*., 2021), and induced stomatal closure (Marrs *et al*., 2020). Recent proximal SIF data have shown and explained linear and nonlinear relationships between SIF and GPP dependent on temporal resolution of the data across a variety of ecosystems (Paul‐Limoges *et al*., 2018; Chen *et al*., 2022; Z. Pierrat *et al*., 2022; Buareal *et al*., 2024).

Despite these nuances, SIF and photosynthesis are mechanistically linked through both shared drivers (PAR and *f*PAR) and photosynthetic links between $\phi_{F}$ and $\phi_{P}$. Proximal remote sensing data have provided evidence that $\phi_{F}$ and $\phi_{P}$ co‐vary over coarse spatiotemporal scales, which strengthens the relationship between SIF and GPP (Magney *et al*., 2020; Z. A. Pierrat *et al*., 2022, 2024b). Thus, by scaling up insights and foundational relationships made at the proximal level to spaceborne data, SIF has a wide variety of applications.

Proximal SIF can be used to understand ecosystem carbon fluxes, track GPP across both seasonal and diurnal scales, and draw more mechanistic linkages between observed SIF and biophysical properties controlling the observation (Yang *et al*., 2015, 2017; Rossini *et al*., 2016; Magney *et al*., 2019; Z. A. Pierrat *et al*., 2022). Proximal SIF has also been used to tie plant carbon uptake with heat/energy dissipation dynamics under cold temperature and light stress by directly comparing proximal SIF to leaf pigment samples within the field of view of the instrument (Magney *et al*., 2019; Raczka *et al*., 2019), an application that is not possible with the coarse spatial resolution of spaceborne SIF data. Although indirectly, SIF can also be used to understand water fluxes and has been used to detect and model transpiration (Lu *et al*., 2018; Shan *et al*., 2019, 2021; Pierrat *et al*., 2021; Nehemy *et al*., 2023). To this end, proximal SIF data can be paired with sap flux or stem radius measurements to more directly tie remote sensing signals with specific water flux or carbon accumulation processes. Early stress detection from heat or drought is also possible with SIF data (Middleton *et al*., 2011; Ač *et al*., 2015; Berger *et al*., 2022; Geng *et al*., 2022; Martini *et al*., 2022; Parazoo *et al*., 2024). Finally, because the SIF signal is sensitive to the light reactions of photosynthesis, SIF has the potential to be used in alternative partitioning approaches to separate net ecosystem exchange (NEE) into GPP and ecosystem respiration (R_eco_), which would require proximal SIF to match the spatiotemporal resolution of flux data (Kira *et al*., 2021; Zhan *et al*., 2022).

### 3. Temperature dependent processes with thermal infrared radiation

Thermal infrared radiation sensors measure thermal energy in the 8–14 μm range (common instruments in Table S2). This energy includes emissions from the target, reflections from the target, and energy attenuated by the atmosphere along the path between the sensor and the target (Aubrecht *et al*., 2016; Johnston *et al*., 2021). Once corrections are applied to isolate the emitted radiation from the object of interest (see Notes S1.3), TIR sensors can be used to retrieve surface temperature using the Stefan–Boltzmann law:

(Eqn 3) $$M=\varepsilon \times \sigma \times T^{4}$$

where *M* is emitted TIR, ε is the target's emissivity (unitless, on a scale from 0–1), σ is the Stefan–Boltzman constant ($\sigma =5.670374419\ldots \times 10^{-8}\;Wm^{-2}K^{-4}$), and *T* is the target's temperature in Kelvin (K, Aubrecht *et al*., 2016). Target emissivity varies with species and ontogeny (Ribeiro da Luz & Crowley, 2007; Richardson *et al*., 2021) and cannot always be easily resolved at large pixel sizes – particularly for mixed pixels. To that end, sampling at a finer spatial scale with proximal (Chen, 2015; Johnston *et al*., 2021) or airborne (Meerdink *et al*., 2019) remote sensing of individual surfaces having different emissivities is helpful.

Leaf and plant temperatures both regulate plant function (e.g. respiration and photosynthetic rates) and are regulated by plant function (e.g. evaporative cooling of the leaf surface); therefore, temperature variations within an image or across time generate insights into eddy‐covariance data (Farella *et al*., 2022). Proximal TIR data can be used to estimate individual tree transpiration using the PT‐JPL algorithm (Fisher *et al*., 2008) and show good agreement between TIR‐derived and eddy‐covariance latent heat fluxes (Javadian *et al*., 2024). This application can reveal interspecies vulnerability to environmental stressors beyond what is observable with eddy‐covariance data alone and has the potential to improve and inform energy balance closures and the partitioning of evaporation and transpiration (Stoy *et al*., 2019) at a fine spatiotemporal resolution (Pierrat *et al*., 2024c). TIR data can further contextualize ecosystem fluxes and vice versa because surface energy fluxes regulate an object's temperature (Still *et al*., 2019). Recent studies have used proximal TIR sensors in conjunction with eddy‐covariance measurements to conduct multi‐site syntheses examining how surface temperature responds to ecosystem fluxes across biomes (Burchard‐Levine *et al*., 2021; Javadian *et al*., 2022; Panwar & Kleidon, 2022).

Proximal TIR data can probe the function of individual plant leaves at a high enough temporal resolution to resolve plant processes, such as phenotypic plasticity, photosynthetic acclimation, leaf water content, stomatal conductance, edge effects, disease detection, stress responses, and diurnal temperature responses (Farella *et al*., 2022). Most photosynthetic reactions are temperature‐dependent and well characterized at the leaf and plant scale; however, the temperature response functions at the canopy/landscape scale integrated over multiple species and functional types are largely unknown or currently in development (Johnston *et al*., 2021). Because leaf temperature exerts an important control on plant carbon fluxes, proximal TIR sensors can help resolve fine‐scale variability in plant carbon and water cycling (Kibler *et al*., 2023; Uni *et al*., 2023). Surface temperatures also allow us to probe plant resilience and vulnerability to extreme environmental conditions. A prime example of this application is discerning whether plants surpass damage‐inducing critical temperature thresholds (Doughty *et al*., 2023; Still *et al*., 2023), but they can also be used to test resilience to drought and water limitations. Thus, TIR measurements are a key tool for model refinement and evaluation, which can only be performed at a proximal scale.

### 4. Biomass and plant water content with microwave backscatter

The microwave region of the electromagnetic spectrum (*c*. 2–30 cm) is most commonly used to track changes in surface water dynamics, including freeze–thaw state (Derksen *et al*., 2017; Roy *et al*., 2020), soil moisture (Larson *et al*., 2008, 2009), snow depth (Larson & Nievinski, 2013), vegetation optical depth (VOD) (Frappart *et al*., 2020; Moesinger *et al*., 2020), vegetation water content (VWC) (Momen *et al*., 2017; Feldman *et al*., 2021), and tipping points in plant mortality (Krishnamurthy *et al*., 2022). Surface water attenuates microwave radiation, and the degree of attenuation can be used to infer changes in its state. Satellite sensors have been collecting data in the microwave region since the late 1970s, making the microwave record one of the longest satellite records available (Smith *et al*., 2019; Moesinger *et al*., 2020).

At the site scale, microwave measurements provide unique insights into both ecosystem structure and water dynamics that help interpret H_2_O, CO_2_, and energy flux terms (common instruments in Table S3). For instance, microwave‐based VOD, or the attenuation of the microwave signal through aboveground vegetation (Brakke *et al*., 1981; Frappart *et al*., 2020), is sensitive to, and uniquely, links ecosystem structure (e.g. biomass) and water dynamics (e.g. VWC) (Baur *et al*., 2019; Humphrey & Frankenberg, 2023; Schmidt *et al*., 2023). VOD has been found to be near‐linearly related to VWC (Jackson & Schmugge, 1991), with the scale factor between the two depending on observation frequency, forest type, and structure (height, biomass density, and gap size). VOD has further emerged as a valuable proxy for plant water potential, offering new avenues for the large scale monitoring of rapid changes in physiological function (Matheny *et al*., 2015; Nolan *et al*., 2020; Konings *et al*., 2021; Dou *et al*., 2023). As such, VOD estimates have been used for a wide variety of applications from quantifying slow changes in aboveground vegetation biomass pools (Hill *et al*., 1999; Liu *et al*., 2015) to rapid rainfall pulse‐driven changes in VWC and leaf water potential (Paloscia *et al*., 2004; Momen *et al*., 2017; Feldman *et al*., 2021; Forkel *et al*., 2023), which may go unresolved with long temporal revisit of spaceborne remote sensing. Due to the inherent link between carbon and water fluxes, VOD has also been utilized as a proxy for GPP (X. Wang *et al*., 2020; Dou *et al*., 2023) and NEE (Feldman *et al*., 2021). Yet, limitations quickly arise when using widely available satellite‐based passive microwave measurements, due to their coarse spatial (9‐ to 36‐km) and temporal (1‐ to 2‐day) resolution.

Tower‐mounted instruments capable of near‐continuous proximal microwave observations at the individual plant scale are an exciting research frontier for: high‐spatiotemporal resolution measurement of soil moisture, VOD, VWC, and plant water status (Holtzman *et al*., 2021; Humphrey & Frankenberg, 2023); and validation for spaceborne soil moisture and VOD observations (Feldman, 2024). When combined with *in situ* measurements of soil moisture, H_2_O, and energy fluxes, high‐frequency, canopy‐scale VOD estimates from near‐surface microwave sensors fill a critical gap in our ability to monitor the full soil–plant–atmosphere water continuum. Furthermore, high‐spatiotemporal VOD estimates from tower‐mounted instruments offer novel insights into plant physiological dynamics, including drought response mechanisms (Frolking *et al*., 2011; Saatchi *et al*., 2013; Rao *et al*., 2019) and plant water status regulation strategies (van Emmerik *et al*., 2015; Schroeder *et al*., 2016; Konings & Gentine, 2017). For instance, proximal microwave sensing has been recently applied to monitor leaf water content diurnal dynamics in a forest in Pasadena, CA, USA (Humphrey & Frankenberg, 2023) and leaf water potential seasonal dynamics in a forest in Ozark, MO, USA (Yao *et al*., 2024). Yet, examples of proximal microwave sensing remain rare in the literature, leaving new explorations, especially those co‐located with field‐based ecophysiological and/or flux tower measurements, ripe with novel lines of scientific inquiry and high likelihood for new discoveries.

### 5. Canopy structure with LiDAR

Terrestrial LiDAR (TLS) is a form of active remote sensing that pulses light at specific wavelengths (typically visible and NIR) to measure the distance between the sensor and a target thereby providing 3D information on canopy structure down to sub‐centimeter precision. Canopy structure, including the spatial arrangement and amount of leaves and woody material, influences absorbed radiation and is thus one of the major drivers of ecosystem fluxes (Eqn 1) and optical remote sensing signals (Verrelst *et al*., 2015; Verbeeck *et al*., 2019; Migliavacca *et al*., 2021). Moreover, the canopy structure itself is an important ecosystem trait that sheds light on the survival and growth strategies of plants (Malhi *et al*., 2018; Yang *et al*., 2023) and for estimating aboveground biomass and carbon storage (Eitel *et al*., 2016). LiDAR is regarded as the most efficient and accurate canopy structure retrieval technique applicable at all scales (ground, air‐, and spaceborne (Asner *et al*., 2008; Calders *et al*., 2020; Dubayah *et al*., 2020)).

Vegetation canopies are often heterogeneous, with different vegetation types, varying vertical profiles (i.e. the number and arrangement of canopy layers), and changing canopy roughness or gap size distribution (Chasmer *et al*., 2011; Zhao *et al*., 2015; Chu *et al*., 2018; Vicari *et al*., 2019; Béland & Baldocchi, 2021; Stovall *et al*., 2021) all of which impact the light absorption in the canopy and subsequent fluxes (Eqn 1). TLS data can help us characterize the vertical profile of a flux footprint and can help us understand the fractional contributions between over‐ and understory elements, which can explain the vertical profile of leaf optical properties related to leaf traits and fluxes. Leaf area index, leaf angle, and clumping also affect canopy fluxes by impacting the radiation distribution within the canopy (Monson & Baldocchi, 2014; Chen, 2018; Yang *et al*., 2023). Changes in leaf area due to environmental (e.g. hurricane) or biological (e.g. spongy moth attacks) factors affect canopy fluxes and can be well captured by repeat TLS (Frolking *et al*., 2009; Atkins *et al*., 2020; Leitold *et al*., 2022) and typically occur at resolutions spaceborne remote sensing cannot capture. Repeat TLS can also provide information on the temporal variation of aboveground biomass, which, coupled with flux tower data, can help us understand carbon assimilation and carbon accumulation and investment in photosynthetic vs nonphotosynthetic components (Calders *et al*., 2015; Eitel *et al*., 2016; Stovall *et al*., 2017, 2018). Finally, LiDAR data have been used to estimate water vapor and latent energy fluxes (Cooper *et al*., 2000), although this application is much less common.

The trend of the increasing fidelity of TLS enables highly detailed 3D representations of canopies (i.e. voxels (Béland *et al*., 2014) and Quantitative Structural Models ‘QSMs’, (Calders *et al*., 2018)). Autonomous *in situ* laser scanners, such as the LEAF (Environmental Sensing Systems, Australia), provide sub‐daily data capturing subtle canopy structure changes not previously possible by commonly used passive optical techniques (e.g. hemispherical photography) (Woodgate *et al*., 2015; Calders *et al*., 2023). When coupled with radiative transfer modeling (RTM), these 3D representations can be used to derive metrics that are not physically observable and are highly synergistic with other forms of remote sensing, such as the SIF escape fraction (*f*_esc_, Eqn 2) (Zeng *et al*., 2019). Overall, high‐resolution LiDAR data reveal physiological and physical processes previously unobservable at coarser spatiotemporal scales, offering numerous applications from local to global ecology (current data available in Table S5).

## III. Synergies

**Fig. 2:**
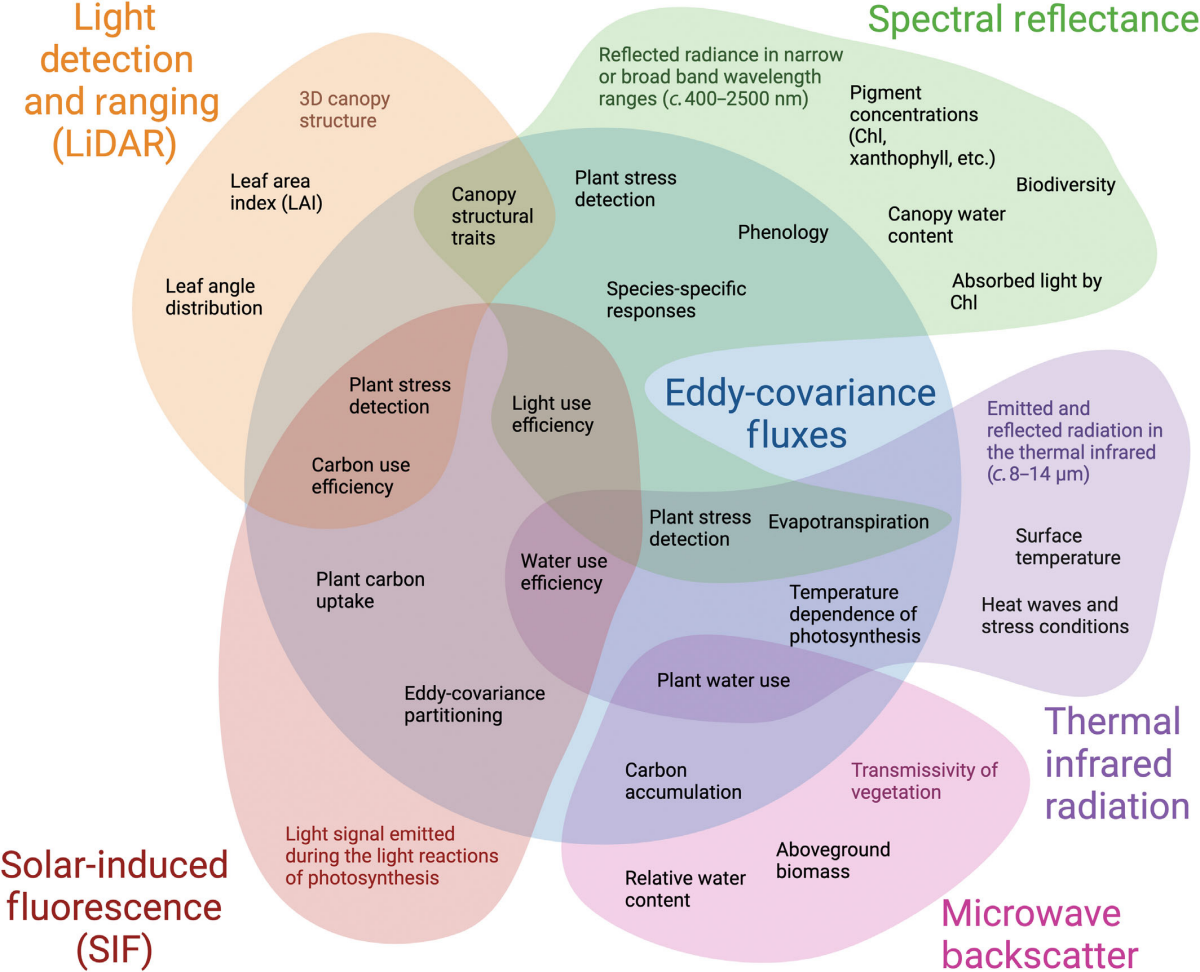
Overview of synergies between proximal remote sensing and eddy‐covariance flux data. Inspired by Smith *et al*. (2019) and Stavros *et al*. (2017) and adapted to focus on synergies with proximal remote sensing and flux data. Created in BioRender. Pierrat, Z. (2025) https://BioRender.com/r49o000.

Combining multiple remote sensing types with ecosystem flux data can open a suite of new parameters for understanding and modeling ecosystems, and their role in the larger Earth system (Fig. 2). Schimel *et al*. (2019) and Stavros *et al*. (2017) have laid out how synergistic spaceborne data can offer new insights into plant function; however, for many of the plant and ecosystem processes spaceborne observations have the potential to observe, there is a need for further uncertainty quantification to distinguish real phenomena from other sources of information. This goal requires the use of proximal sensing to establish mechanistic links between remote sensing signals and plant processes, which can then feed into further algorithm development, model improvements, and data integration.

Combining spectral reflectance with SIF data enhances our ability to predict plant carbon uptake because reflectance provides contextual information on plant LUE and structural parameters impacting the SIF signal (Dechant *et al*., 2020; Z. A. Pierrat *et al*., 2022; Zhang *et al*., 2022). These multiple signal sources have only recently been able to be teased out using proximal data and are ready to be applied to spaceborne observations.

Combining thermal (related to evapotranspiration) and microwave (related to aboveground water content and biomass) can be used to infer plant water use and water use strategies. This is especially useful in combination with *in situ* measurements of soil moisture and humidity, which would allow tracking of water transport along the full soil–plant–atmosphere continuum and must be tested at the site level before scaling across space and time. Linking metrics of carbon uptake (SIF or spectral reflectance) with water loss (TIR and microwave) can help us understand plant water use efficiency, which is highly dynamic across diurnal and seasonal scales. Additionally, we could observe and test relationships between increasing heat (canopy temperatures from TIR) and plant health (canopy nutrient contents derived from spectral reflectance), or between heat and ecosystem function (SIF) or water use (evapotranspiration, VWC, SIF) (Martini *et al*., 2022).

In many cases, interpreting other types of optical signals and connecting to fluxes requires a detailed characterization of canopy structure, including the spatial arrangement and density of leaf and wood scattering elements (Myneni *et al*., 1986; Verrelst *et al*., 2015; Chen, 2018; Magney *et al*., 2020) and the distance between the target and sensor. Combining LiDAR with spectral reflectance, SIF, or thermal, can help us determine what parts of the optical signal are driven by physiological vs structural change (Stovall *et al*., 2018). Nonphotosynthetic vegetation (e.g. branches and stems) and soil background have non‐negligible impacts on measured radiance (Malenovský *et al*., 2021; Zeng *et al*., 2022). Canopy shading can also cause up to a 50% reduction in SIF and reflectance quantities due to normalization using hemispherical irradiance measured at the top of the canopy (Damm *et al*., 2015). Coupling high‐resolution canopy structures derived from TLS with RTM (Magney *et al*., 2016) to simulate the impact of vegetation structure and composition on optical signals can help disentangle these physical from physiological drivers of fluxes and remote sensing signals (Verrelst *et al*., 2010; Regaieg *et al*., 2021). To this end, future development of multi‐ and hyperspectral TLS offers exciting opportunities to address these research directions directly and enhance TLS information by distinguishing between vegetated and nonvegetated signals (Eitel *et al*., 2016; Calders *et al*., 2020). The target‐to‐sensor range information provided by LiDAR is particularly important for TIR and SIF atmospheric corrections. SIF retrieval methods that are sensitive to oxygen absorption depend on path length and will need to be corrected for atmospheric effects (van der Tol *et al*., 2023). This correction is pertinent for imagery and multi‐angular sensors where path lengths (sensor to target range) change substantially (Grossmann *et al*., 2018; Woodgate *et al*., 2020). Finally, broadband TIR will be more impacted than narrowband TIR due to water content in the atmospheric column (Guillevic *et al*., 2018) and can be adjusted with LiDAR.

These examples represent just a few of the potential insights that can be gained by combining multiple types of proximal remote sensing with flux and site‐level data. A single site fully equipped to measure fluxes as well as spectral reflectance, SIF, TIR, microwave backscatter, and LiDAR would be able to comprehensively observe ecosystem structure and function and provide insights into complex relationships between predicted changes in climate and ecosystem health.

## IV. The case for a network

A coordinated network of proximal remote sensing instruments offers multiple benefits to advance flux and ecosystem science. A prime successful example of this coordination is the PhenoCam Network (https://phenocam.nau.edu/webcam/), which is a network of RGB cameras with a standardized data processing framework to estimate the onset and cessation of greenness from over 700 sites (Richardson *et al*., 2018; Seyednasrollah *et al*., 2019). PhenoCam has led to significant advancements not only in phenological research but also in understanding the interplays between phenology, ecosystem fluxes, and environmental feedbacks (Richardson, 2023). Building on decades of progress in proximal instrumentation, the spectral techniques outlined in this review offer significant potential for further advancements and coordination.

At flux sites, accurately partitioning NEE into R_eco_ and GPP is important for understanding the terrestrial carbon cycle and future climate projections. However, the standard partitioning methods, for example nighttime (Reichstein *et al*., 2005) and daytime partitioning (Lasslop *et al*., 2010), rely on simplified empirical models, which may lead to either overestimations of daytime total ecosystem respiration or underestimations of nighttime respiration if leaf‐level inhibition occurs (Wehr *et al*., 2016; Keenan *et al*., 2019). Using remote sensing to represent photosynthesis and photosynthetic properties may present new avenues for the partitioning of NEE (Zhan *et al*., 2022; Chen *et al*., 2024), although cross‐compatibility of remotely sensed data is essential for this to work at scale.

Next, filling long‐term gaps (weeks to months) in eddy‐covariance data is particularly challenging due to the potential changes in underlying ecosystem properties over time, as well as the errors and uncertainties associated with gap‐filling algorithms (Richardson & Hollinger, 2007). The most common gap‐filling approach, marginal distribution sampling (MDS), still does a poor job in filling extra‐long gaps and can create systematic bias in carbon balance estimates (Vekuri *et al*., 2023). By instrumenting flux towers with proximal remote sensing, we can develop new approaches for gap filling and use these synergistic datasets as a fail‐safe when one instrument is not working properly. Combining proximal remote sensing data with footprint climatology also results in a comprehensive dataset that offers more detailed insights into vegetation structure, topography, and potential species‐specific source/sink effects on the observed fluxes. For example, by combining proximal remote sensing and flux data, we can improve our understanding of the timing and drivers of phenological events at an individual species level (Pierrat *et al*., 2021; Moon *et al*., 2022), which is not discernible from spatially averaged flux or satellite data alone. This integration of information has been shown to significantly enhance the analysis and interpretation of flux data (Kljun *et al*., 2015; Chu *et al*., 2021; Holtzman *et al*., 2021) and can be implemented into model frameworks to improve representation of key processes.

Beyond advancing site‐level flux science, an investment in proximal remote sensing is increasingly relevant for new and upcoming satellite missions which provide synergistic observations of ecosystem processes (Stavros *et al*., 2017). This is particularly relevant as proximal remote sensing data can be recorded even under cloudy sky conditions. These observations will shed light on the connections between biologic processes and optical observations under direct vs diffuse illumination conditions that go unobserved by passive spaceborne observations. Existing co‐located observations on the International Space Station include (among others), the Orbiting Carbon Observatory (OCO) 3 measuring SIF, the ECOsystem Spaceborne Thermal Radiometer Experiment on Space Station (ECOSTRESS) deriving land surface temperature and emissivity with TIR, the Global Ecosystem Dynamics Investigation (GEDI) deriving canopy height and internal structure with LiDAR, and the Earth Surface Mineral Dust Source Investigation (EMIT) measuring spectral reflectance (Stavros *et al*., 2017; Xiao *et al*., 2021). Future satellite missions as part of NASA's Earth System Observatory (Space Studies Board *et al*., 2019) will include biomass estimates from the NISAR mission and spectral reflectance and TIR data from the Surface Biology and Geology (SBG) mission and will be complimented by European Space Agency's FLuorescence EXplorer (FLEX) measuring SIF and spectral reflectance (Drusch *et al*., 2017). These represent just a few of the existing and upcoming missions, which will continue synergistic observations of the Earth system enabling new insight into plant functioning.

Proximal remote sensing (either co‐located on flux towers or independent) has already proven useful for the calibration, validation, and evaluation of existing spaceborne measurements (Parazoo *et al*., 2019; Hu *et al*., 2022; Feldman, 2024). The PRecursore IperSpettrale della Missione Applicativa (PRISMA) mission from the Italian Space Agency has demonstrated particular success in this arena with a dedicated field campaign linking proximal, aircraft, and spaceborne data with remarkably close agreement despite limited geographic and temporal coverage (Cogliati *et al*., 2021). Proximal data harmonization will help expand these efforts, ultimately improving confidence in spaceborne data. An investment in proximal remote sensing should also be considered for sites located in the global south due to the high variety of ecosystem types and their potential for carbon sequestration; and there remains a lack of *in situ* measurement systems available. Proximal remote sensing can be considered as an option in locations where eddy‐covariance flux observations are not possible due to local topography or meteorological conditions. The increased spatiotemporal resolution provided by a network of proximal sensors can support the harmonization of multiple instruments, interpretation and downscaling of spatially averaged, snapshot in time, spaceborne observations, and reveal new ecological insights on mechanistic drivers of observed signals not captured by space‐based data. Finally, sites equipped with multiple types of proximal remote sensing instruments could be used as a low‐cost testbed for algorithm development for upcoming air‐ and spaceborne missions, enabling us to test design parameters at an even higher spatiotemporal resolution than currently possible (Cawse‐Nicholson *et al*., 2022).

## V. A path forward for networking proximal remote sensing data

As the utility of various proximal sensing methods has been shown, there are critical shortcomings in data integration. At present, we have a highly fragmented ecosystem in terms of instrumentation, data management, and data processing, which limits the easy integration of various data streams, within and between methods and external data sources (e.g. flux measurements or satellite remote sensing data). For example, there are presently no widely accepted standards of practice for collecting proximal spectral reflectance or SIF data in the literature. This inconsistency hinders the ability to consistently apply techniques and insights gleaned at one site more broadly for understanding global change biology. Recent and rapid advances in the field have led to frequent changes in available instrumentation and challenges creating continuity among datasets (Notes S1). Previous attempts to address this issue have largely come from organizations, such as EUROSPEC (Porcar‐Castell *et al*., 2015), and international collaborations, such as SpecNet (Gamon *et al*., 2006, 2010). These efforts have been successful in outlining general guidance for long‐term proximal remote sensing (Notes S1, Porcar‐Castell *et al*., 2015), but have struggled to develop a coordinated network. This can be attributed to: disparate sensors and methods driving the production of unique datasets that cannot readily be put into a single database (i.e. a lack of standardization); and a lack of sufficient sustained funding to help maintain a database of products and tools and update protocols based on available instrumentation.

Despite the aforementioned challenges, there have been successful efforts to coordinate proximal remote sensing datasets across sites (summarized in Notes S1–S3). Arguably, the most successful of these efforts has come from the PhenoCam Network. The PhenoCam's success can be attributed to: its close integration with existing flux networks; ease of data access; and coherent postprocessing. All software, (i.e. the phenocam R package (Hufkens *et al*., 2018)) and python and hardware specifications (Seyednasrollah *et al*., 2019) are openly available. PhenoCam can serve as a prime example of the utility of networked proximal remote sensing and a resource for how to generate such a network (Richardson, 2023).

Realizing the full potential of proximal remote sensing hinges on the community's ability to develop standardized measurement, deployment and processing techniques, comprehensive metadata documentation, and active participation in collaborative networks. These efforts will enable the consolidation and integration of different data sources, ultimately allowing us to use these data across sites for answering grand challenges in global ecology (Schimel *et al*., 2019). As a first step in this direction, we provide the status of sensor based best practices (i.e. configuration and calibration) for spectral reflectance, SIF, TIR, microwave, and LiDAR (Notes S1–S3). For proximal remote sensing types with more developed standards of practice, recent efforts have made progress on consolidating proximal remote sensing data into publicly available databases, although much of this work is ongoing and rapidly evolving. To facilitate data use and promote adoption of existing databases, we provide the status of data availability, including existing, growing, and evolving data networks (Notes S3). These coordinated efforts will help to democratize proximal remote sensing techniques, making them more accessible to the scientific community and encouraging the widespread adoption of recommended sensors, best practices, and metadata. With the recent developments in proximal remote sensing technology, rapid proliferation of satellite instrumentation, and community initiatives, such as the AmeriFlux Year of Remote Sensing, the time is now apt to carry this work forward (Pierrat *et al*., 2023). Our report, as presented here, is a critical and timely step in this direction, bringing together a comprehensive overview of available data, key voices, and expertise.

## VI. Conclusions

Proximal remote sensing data have demonstrated the potential to considerably advance Earth system science by linking observations across scales (from the site to the globe and from minutes to days), shedding light on key ecological processes that are otherwise unobserved, and providing mechanistic insight into the physical and physiological drivers of observed fluxes. We highlighted key areas where spectral reflectance, SIF, TIR, microwave backscatter, and LiDAR can be used (on their own and in combination with each other) in conjunction with flux data to better understand ecosystem processes and synthesize recent advances in ecosystem and flux science using these data. Specifically, we discuss how each measurement can address questions related to the scale dependence of ecosystem processes, physical and biological drivers of ecosystem processes and observations, and how synergistic observations provide a more complete picture of plant and ecosystem science, and ultimately, global change biology. We also outlined best practices for those interested in getting started with proximal remote sensing and provided resources for individuals to find existing data sources as a first step toward building a more coordinated network. Our aim was to make proximal remote sensing data streams more widespread, more accessible, and more information‐rich to facilitate global change biology research. This review is an essential step toward growing an open dialogue for consistent acquisition and processing of proximal remote sensing data for these applications. Expanding the availability and accessibility of proximal remote sensing will help facilitate the use of these data for advancing Earth system science now and into the future.

## Competing interests

None declared.

## Author contributions

AU, BRKR, BEM, CD, CLK, CYSW, GK, JD, KM, KH, MB, MPD, MRJ, TJ, RSM, WPR, WKS, WW, XY, YRSG, YL and ZAP contributed to the conceptualization. LPA, TJ, TSM, WPR, WW, XY and ZAP contributed to the methodology. ZAP contributed to the investigation. AU, BEM, CD, CLK, GK, GT, JLD, KM, MA, MRJ, MJ, TSM, WPR, WKS, WW, XY, YRSG and ZAP contributed to the visualization. JLD, KCN, TSM, WPR, WW, XY and ZAP contributed to the supervision. ALDS, AELS, BRKR, BEM, CD, CLK, CYSW, GK, GT, JLD, JM, KM, LPA, MB, MPD, MRJ, MJ, TSM, WPR, WKS, WW, XY, YRSG, YL and ZAP contributed to the writing – original draft. AU, ALDS, AELS, BRKR, BEM, CLK, CYSW, GK, GT, JLD, JAG, JBF, JKM, KCN, KM, KH, LPA, MB, MPD, MA, MRJ, MJ, TJ, TSM, WPR, WKS, WW, XY, YRSG and ZAP contributed to the writing – review and editing.

## Disclaimer

The New Phytologist Foundation remains neutral with regard to jurisdictional claims in maps and in any institutional affiliations.

## Supporting information


**Notes S1** Recommended Best Practices for Proximal Remote Sensing provides guidance on instrument setup, retrievals, temporal aggregation, calibrations, metadata, and support data for spectral reflectance, solar‐induced fluorescence, thermal infrared radiation, microwave, and LiDAR.
**Notes S2** Metadata recommendations for tower‐mounted hyperspectral and SIF instruments provides example metadata formatting to facilitate the cross‐compatibility of these data and reporting standards.
**Notes S3** Existing Publicly Available Data provides the status of proximal remote sensing data currently published following FAIR data principles.
**Table S1** Instrument descriptions for proximal spectral reflectance and SIF.
**Table S2** Instrument descriptions for proximal thermal infrared radiation.
**Table S3** Instrument descriptions for proximal microwave measurements.
**Table S4** Overview of existing publicly available proximal SIF datasets and a link to a more updated database.
**Table S5** Selection of available TLS datasets.
Please note: Wiley is not responsible for the content or functionality of any Supporting Information supplied by the authors. Any queries (other than missing material) should be directed to the *New Phytologist* Central Office.

## Data Availability

Refer to Notes S1–S3 and Tables S1–S5 for currently published data in the field of proximal remote sensing.
