# Supplementary material for: Proximal remote sensing: an essential tool for bridging the gap between high‐resolution ecosystem monitoring and global ecology
Source: New Phytol. 2025 Jan 23;246(2):419–36. doi: 10.1111/nph.20405 (PMC11923411; doi:10.1111/nph.20405)
Supplement: Supplementary file 1 — Notes S1 Recommended Best Practices for Proximal Remote Sensing provides guidance on instrument setup, retrievals, temporal aggregation, calibrations, metadata, and support data for spectral reflectance, solar‐induced fluorescence, thermal infrared radiation, microwave, and LiDAR. Notes S2 Metadata recommendations for tower‐mounted hyperspectral and SIF instruments provides example metadata formatting to facilitate the cross‐compatibility of these data and reporting standards. Notes S3 Existing Publicly Available Data provides the status of proximal remote sensing data currently published following FAIR data principles. Table S1 Instrument descriptions for proximal spectral reflectance and SIF. Table S2 Instrument descriptions for proximal thermal infrared radiation. Table S3 Instrument descriptions for proximal microwave measurements. Table S4 Overview of existing publicly available proximal SIF datasets and a link to a more updated database. Table S5 Selection of available TLS datasets. Please note: Wiley is not responsible for the content or functionality of any Supporting Information supplied by the authors. Any queries (other than missing material) should be directed to the New Phytologist Central Office. [file NPH-246-419-s001.pdf]

New Phytologist

**_New Phytologist_ Supporting Information**

Article title: Proximal remote sensing: an essential tool for bridging the gap between high resolution ecosystem monitoring and global ecology

Authors: Zoe Amie Pierrat[1*], Troy S. Magney[2], Will P. Richardson[3], Benjamin R. K. Runkle[3], Jen L. Diehl[4,5], Xi Yang[6], William Woodgate[7,8], William K. Smith[9], Miriam R. Johnston[10], Yohanes R. S. Ginting[11], Gerbrand Koren[12], Loren P. Albert[13], Christopher L. Kibler[14], Bryn E. Morgan[14], Mallory Barnes[15], Adriana Uscanga[16], Charles Devine[9], Mostafa Javadian[4], Karem Meza[17], Tommaso Julitta[18], Giulia Tagliabue[19], Matthew P. Dannenberg[10], Michal Antala[20], Christopher Y.S. Wong[21], Andre L. D. Santos[22], Koen Hufkens[23], Julia K. Marrs[24], Atticus E. L. Stovall[25], Yujie Liu[4], Joshua B. Fisher[26], John A. Gamon[27], Kerry Cawse-Nicholson[1]; [1]Jet Propulsion Laboratory, California Institute of Technology, Pasadena, CA, USA [2]Department of Plant Sciences, University of California, Davis, CA, USA [3]Biological & Agricultural Engineering, University of Arkansas, Fayetteville, AK, USA [4]Center for Ecosystem Science and Society, Northern Arizona University, Flagstaff, AZ, USA [5]School of Informatics, Computing & Cyber Systems, Northern Arizona University, Flagstaff, AZ, USA [6]Department of Environmental Sciences, University of Virginia, VA, USA [7]School of the Environment, The University of Queensland, 4072, Australia [8]CSIRO, Space and Astronomy, Kensington, 6151, WA, Australia [9]School of Natural Resources and the Environment, University of Arizona, Tucson, Arizona, USA [10]Department of Geographical and Sustainability Sciences, University of Iowa, Iowa City, IA, USA [11]Climate Monitoring Group, Department of Meteorology, Institute of Geosciences, University of Bonn, 53121 Bonn, Germany [12]Copernicus Institute of Sustainable Development, Utrecht University, Utrecht, The Netherlands [13]Forest Ecosystems & Society, Oregon State University, 321 Richardson Hall, Corvallis, OR, USA [14]Department of Geography, University of California, Santa Barbara, CA, USA [15]O'Neill School of Public and Environmental Affairs, Indiana University, Indiana, USA [16]Department of Geography, Environment, and Spatial Sciences, Michigan State University, East Lansing, MI, USA [17]Department of Civil and Environmental Engineering, Utah State University, Logan, UT, USA [18]JB Hyperspectral Devices, Düsseldorf, Germany [19]University of Milano - Bicocca, Milano, Italy [20]Laboratory of Bioclimatology, Department of Ecology and Environmental Protection, Poznan University of Life Sciences, Poznan, Poland [21]Forestry and Environmental Management, University of New Brunswick, Fredericton, NB, Canada [22]Climate & Ecosystem Sciences Division, Lawrence Berkeley National Laboratory, Berkeley, CA, USA [23]Institute of Geography, University of Bern, Bern, Switzerland; Oeschger Centre for Climate Change Research, University of Bern, Bern, Switzerland [24]National Institute of Standards and Technology, 100 Bureau Drive, Gaithersburg, MD, USA [25]NASA Goddard Space Flight Center, 8800 Greenbelt Rd, Greenbelt, MD, USA [26]Schmid College of Science and Technology, Chapman University, 1 University Drive, Orange, CA, 92866, USA [27]CALMIT, School of Natural Resources, University of Nebraska – Lincoln, Lincoln, NE, USA; *Corresponding Author: Zoe Amie Pierrat, zoe.a.pierrat@jpl.nasa.gov, zpierrat@gmail.com

Article acceptance date: 02 January 2025

The following Supporting Information is available for this article:

**Notes S1:** Recommended Best Practices for Proximal Remote Sensing provides guidance on instrument setup, retrievals, temporal aggregation, calibrations, metadata, and support data for spectral reflectance, solar-induced fluorescence, thermal infrared radiation, microwave, and LiDAR.

**Notes S2:** Metadata recommendations for tower-mounted hyperspectral and SIF instruments provides example metadata formatting to facilitate the cross-compatibility of these data and reporting standards.

**Notes S3:** Existing Publicly Available Data provides the status of proximal remote sensing data currently published following FAIR data principles.

**Table S1:** Instrument descriptions for proximal spectral reflectance and SIF.

**Table S2:** Instrument descriptions for proximal thermal infrared radiation.

**Table S3:** Instrument descriptions for proximal microwave measurements.

**Table S4:** Overview of existing publicly available proximal SIF datasets and a link to a more updated database.

**Table S5:** Selection of available TLS datasets.

**Supplementary Text**

## Notes S1. Recommended Best Practices for Proximal Remote Sensing

Deriving spectral reflectance requires a measurement of the reflected radiation (outgoing) and irradiance from the sun (incoming). Dividing reflected radiation by incoming irradiance at each wavelength gives a plant reflectance value (as a percent or decimal percent, 0-1). Measuring changes in plant reflectance across the visible (VIS, 400-700 nm), near-infrared (NIR, 700-1100 nm), and short-wave infrared (SWIR, 1100 nm - 2500 nm) regions has enabled estimation of vegetation traits and functions across a wide range of scales (Figure 1, (Ustin et al., 2004; C. Y. S. Wong, 2023)).

### Notes S1.1. Spectral Reflectance Best Practices

Within spectral reflectance research, there are both multispectral systems (that use a few bands) and hyperspectral systems (which measure contiguous discrete wavelengths across the VIS-NIR spectrum, typically ~400 to 1000 or 2500 nm spectral range). These systems are summarized in Table S1.

The most widespread tower multispectral data comes from the PhenoCam network, a network of over 700 sites, and an image archive of over 60 million pictures (Richardson, 2023;

Richardson et al., 2018). The majority of these images are collected every 30-minutes and provide data in the red, blue and green parts of the spectrum (including some with an NIR band), which has enabled widespread monitoring of leaf phenology (timing of leaf on and leaf off) and also more nuanced photosynthetic phenology in evergreen and deciduous species, due to the sensitivity of the green part of the spectrum to changes in carotenoid pigments (Filippa et al., 2018; Y. Liu et al., 2020; Seyednasrollah et al., 2020).

The simplest multispectral sensors (beyond RGB) that have been placed on towers have been made from LEDs or photodiodes and have both outgoing (upwelling) and incoming (downwelling) sensors. These can be used to derive vegetation indices (such as the Normalized Difference Vegetation Index (NDVI) and Photochemical Reflectance Index (PRI)) to track seasonal changes in plant greenness, stress, and light-use efficiency (J. A. Gamon et al., 2015; Garrity et al., 2011; Harris et al., 2014; Magney, Eitel, et al., 2016; Magney, Vierling, et al., 2016; Ryu et al., 2010; C. Y. S. Wong et al., 2020; J. C. Yang et al., 2020). These systems have been built using off-the-shelf components (Garrity et al., 2011; Ryu et al., 2010), but have also been produced more widely by companies such as Decagon Devices (now METER), Apogee Instruments, and Skye Sensors (~ $200-300 USD, Table S1). While the aforementioned instruments are available at the time of this publication, these instruments have developed alongside their scientific applications, leading to a high turnover of available instrumentation. Another primary challenge with these low-cost systems is cross calibration, which can be difficult in environments which experience a wide range in environmental conditions, as temperature extremes and  moisture can readily alter the function of sensors (J. A. Gamon et al., 2015). However, since the amount of upwelling and downwelling energy is recorded as a digital number, and divided to get reflectance, radiometric calibration is not needed - so long as cross-calibration between the upward and downward sensors is performed (J. A. Gamon, 2015). Finally, temporal drift is common among these low-cost instruments and should be routinely recalibrated to correct for this.

Hyperspectral sensors have also been placed on towers, typically measuring canopy reflectance in the 400-1000 nm range (Campbell et al., 2019; Grossmann et al., 2018; Hamada et al., 2020; Hilker et al., 2007; Tømmervik et al., 2023; C. Y. S. Wong et al., 2023; Woodgate et al., 2020). These hyperspectral instruments have most commonly been ‘point sensors’, rather than imagers (with exceptions (Woodgate et al., 2020)), and often have a narrow field of view with either ‘scanning’ capabilities – placed on a pan tilt unit, or with internal motors to change the viewing geometry – or are on a mobile platform to sample a larger portion of the flux footprint (J. A. Gamon et al., 2006). Many of these instruments make measurements using the same spectrometer for both radiance and irradiance measurements, whereby the foreoptic points towards the sun underneath a cosine diffuser, or irradiance is measured by down-looking foreoptic using a mobile reference panel that frequently moves beneath the foreoptic (Hamada et al., 2020). If separate spectrometers are used for upwelling and downwelling radiation, frequent radiometric calibration of the spectrometer is critical – thus, it is highly recommended for tower hyperspectral measurements to be made using a single spectrometer. Additionally, detectors must be carefully spectrally and temporally matched to facilitate intercomparison. The development and more widespread adoption of the FloX and RoX systems (JB Hyperspectral Devices, Germany) have presented a significant advancement in creating a network, as these instruments have a standard design, sampling protocol, and processing software (Julitta et al., 2017).

There are presently no widely accepted standards of practice for collecting proximal spectral reflectance data in the literature. Many of these can be manufacturer specific but recent advances in the field have led to frequent changes in available instrumentation and challenges creating continuity among datasets. Previous attempts to address this have largely come from organizations such as EUROSPEC (Porcar-Castell et al., 2015), which was later moved under the umbrella of the larger network SpecNet (J. Gamon et al., 2006; J. A. Gamon et al., 2010). These efforts have been successful in outlining general guidance for long-term proximal remote sensing (Porcar-Castell et al., 2015), but have struggled to develop a coordinated network. This can largely be attributed to a lack of sustained funding which would help maintain a database of products and tools and update protocols based on available instrumentation. The following sections outline key considerations and recommendations for collecting spectral reflectance within the flux tower footprint based on existing literature, and advancements made since the publication of guidance from SpecNet and EUROSPEC.

***Instrument setup***

Selection of instrumentation and viewing geometry should be guided by the characteristics of the eddy-covariance flux footprint at a given site. Although eddy-covariance footprints are orders of magnitude larger than proximal spectral reflectance observations for most configurations, the optical footprint may be representative of the eddy-covariance footprint depending on the heterogeneity of the site and other factors such as meteorological conditions (Chu et al., 2021; J. A. Gamon, 2015; G. Wu et al., 2023). Users are faced with several choices in instrument configuration such as bi-hemispherical vs. hemispherical-conical, nadir vs. off-nadir, and stationary vs. scanning (Sun, Wen, et al., 2023); while trade-offs are inevitable in these decisions, each can be leveraged to improve compatibility between optical and eddy-covariance footprints.

In a relatively homogeneous landscape like a grassland or cropland, deploying instrumentation with a wide fixed field of view (FOV) may be most advantageous (Tømmervik et al., 2023). With fixed instrumentation, the vertical viewing direction should be as close to nadir as possible to minimize directional effects. In a more complex ecosystem, where more than one species is of interest, or where background effects should be minimized, a narrow FOV scanning system or mobile platform will be most useful (Grossmann et al., 2018; C. Y. S. Wong et al., 2023). While this type of instrumentation has the added complexity of angular issues resulting from changing viewing geometry, it enables more mechanistic understanding of components driving the spectral signal (Z. Pierrat et al., 2021). Angular issues and directional effects can also have a significant impact on spectral reflectance (Bai et al., 2023; Jafarbiglu & Pourreza, 2023), therefore, careful documentation of viewing direction and potential shadowing effects (i.e., from the tower itself) is important. For narrow FOV systems, it is additionally important to characterize the exact captured region. This can be accomplished by shining a white LED through the fiber optic in reverse at night, which illuminates the ecosystem component measured at a given viewing direction (Grossmann et al., 2018; Magney, Bowling, et al., 2019; Z. Pierrat et al., 2021) or having a co-located RGB image taken with each retrieval (T. S. Jones et al., 2023; C. Y. S. Wong et al., 2023). Co-located RGB imagery has the additional benefit of providing information on sunlit/shaded portions of the canopy which can aid in signal interpretation. Finally, recent advances in footprint analysis of eddy-covariance data (Chu et al.,

2021), enables users to better spatially match remote sensing observations with eddy-covariance data (Metzger et al., 2013).

***Temporal Aggregation***

Spectral reflectance measurements can be made as frequently as several times per minute; therefore, data producers should take care to aggregate data into a standardized format to make data cross comparable. To match the temporal resolution of flux data, we recommend using the same averaging technique as AmeriFlux and FLUXNET, which typically use 15 minutes on either side of the reported timestamp to report data every 30 minutes (Pastorello et al., 2020). If the instrument has scanning capabilities, the user should ensure a complete scan occurs within that time frame, so the reported data is not biased towards certain ecosystem components. Since irradiance measurements typically are not made simultaneously with reflected radiance measurements, we recommend taking both irradiance and reflected radiance measurements < 5 minutes (in clear sky conditions) apart to ensure near-identical sky conditions. Under unstable (cloudy) conditions, users can also consider using higher temporal resolution (1 second) PAR data to filter data under unstable sky conditions (Z. Pierrat et al., 2021) or using 'sandwich' style data acquisition where a reference is taken before and after to assess stability (Cogliati et al., 2015; Meroni et al., 2011).

***Calibrations***

For multi-site comparisons of hyperspectral reflectance data, proper calibration is key as improper calibration can lead to unintended differences in the retrieved signal (Cendrero-Mateo et al., 2019; Chang et al., 2020). Users should calibrate the data for detector non-linearity, dark current and offsets, stray light, wavelength, FOV (in mobile or scanning systems), and perform radiometric calibrations. Radiometric calibrations should be performed several times per year, unless spectrometers are stored in a temperature-controlled box, in which case calibration should remain stable for several years (Grossmann et al., 2018). More complete details for calibration approaches are published elsewhere (Grossmann et al., 2018; Woodgate et al., 2020). Since the frequency of calibration requirements may vary with instrument, environmental conditions and purpose, a method of checking calibrations in the field would be very helpful in insuring data quality. A future networking initiative could develop a calibration kit for tower based hyperspectral systems analogous to AmeriFlux's Portable Eddy Covariance System (PECS) used for cross-site calibration (Ocheltree & Loescher, 2007).

***Metadata***

To help users better facilitate both the collection and sharing of hyperspectral data, we have developed a standardized set of metadata that should be documented and published with data. This set of metadata is being integrated into the FLUXNET network and is cross-compatible with the FloX system (JB Hyperspectral Devices, Germany). Included in the metadata are features such as spectral range and resolution, FOV and directional information, calibration types and frequency, among other things. The metadata suggestions can be found in Notes S2.

**Notes S1.2. Solar-Induced Fluorescence Best Practices**

The detection of SIF from spaceborne platforms in 2007 (Guanter et al., 2007) spurred a boom of technological development to build tower and ground-based instrumentation for the passive monitoring of SIF (Daumard et al., 2010; Du et al., 2019; Grossmann et al., 2018; X. Yang et al., 2018). We refer readers to (Sun, Wen, et al., 2023) and (Mohammed et al., 2019) for a more detailed overview of existing SIF instrumentation. The key factors preventing intercomparison among published SIF data can be summarized as: 1) FOV and configuration differences, 2) SIF retrieval differences, and 3) pre and post-processing differences (e.g., in calibration standards, frequency of observations, and temporal averaging) (Chang et al., 2020; Julitta et al., 2016; Marrs et al., 2021; R. Wang et al., 2022). Here we present primary considerations and best practices for SIF data collection.

***Instrument setup and temporal aggregation***

Many SIF instruments are combined SIF and hyperspectral sensors or have physical configurations very similar to hyperspectral instruments with a higher spectral resolution. Therefore, we refer readers to Notes S1.1. for guidance on instrument setup and temporal aggregation as these will be consistent between SIF and hyperspectral systems. In addition, as SIF is an emitted signal, it must be isolated from the overall light measured by the instrument and post-processing to separate physical and physiological drivers to link SIF to plant physiology. Therefore, we suggest further calibrations, additional metadata (S2), and additional support data to improve the cross-compatibility of SIF observations across sites.

***SIF Retrievals***

Spectra collected by SIF instruments are the sum of reflected radiation and SIF; therefore, SIF must be isolated or “retrieved” from the vegetation spectra. Due to the relatively weak nature of the signal, SIF can most easily detected and retrieved in narrow solar (Fraunhofer line) and atmospheric absorption (oxygen or water) lines, which are dark compared to radiance in neighboring regions of the spectrum (Frankenberg & Berry, 2017; Meroni et al., 2009; R. Wang et al., 2022). Retrieval methods generally fall into four categories: Fraunhofer line depth (FLD) approaches, spectral fitting methods (SFM), machine learning approaches, and differential optical absorption spectroscopy (DOAS). FLD methods, including the classic FLD (Plascyk, 1975), 3FLD (Maier et al., 2004), improved FLD (iFLD) (Alonso et al., 2008), and Spectral Shape Assumption FLD (SSA-FLD) (N. Wang et al., 2021) use the upwelling radiance within the absorption band and just outside the absorption band to estimate SIF as the degree to which it fills in the absorption line. Each of the FLD methods is built on assumptions about the shape of the reflectance and fluorescence spectra, with iFLD and SSA-FLD containing the most complex assumptions and also being the most robust (Cendrero-Mateo et al., 2019). In contrast, SFMs model the observed upwelling radiance as the sum of reflectance and fluorescence, which are each described by a mathematical function of wavelength (Meroni et al., 2009, 2010); SIF at a given wavelength (typically the center of the absorption band) is then estimated using the fitted parameters. Early work indicated that linear functions were suitable for modeling both reflectance and fluorescence (Meroni et al., 2010), but more recent efforts have improved SFMs by using more complex Gaussian and Voigt functions to model fluorescence (Cogliati et al., 2015). As an alternative, several retrieval methods leverage

machine learning approaches such as singular vector decomposition and partial least squares regression to retrieve SIF after being trained on solar irradiance and non-vegetation spectra (Guanter et al., 2013; Naethe et al., 2022; Scodellaro et al., 2022). DOAS focuses on solar Fraunhofer lines (Figure 1) and leverages changes in line depth to estimate SIF (Grossmann et al., 2018). Because the full SIF spectrum carries more information than SIF retrieved in absorption bands (Magney, Frankenberg, et al., 2019) several methods have been developed to reconstruct the full SIF spectrum, such as the Fluorescence Spectrum Reconstruction (FSR) (Zhao et al., 2014), advanced FSR (aFSR) (Zhao et al., 2018), the Full-spectrum Spectral Fitting (F-SFM) (X. Liu et al., 2015), or SpecFit algorithm (Cogliati et al., 2019). SIF retrievals are an active area of research with no community consensus on which approach is best. Because of this, intercomparisons among retrieval approaches (Chang et al., 2020) are particularly valuable and needed across a wide range of ecosystems and meteorological conditions. We refer the reader to (Cendrero-Mateo et al., 2019) and (Chang et al., 2020) for more details on SIF retrieval methods and their application.

***Calibrations***

Much like hyperspectral measurements, we recommend users calibrate for detector non-linearity, dark current and offsets, stray light, wavelength, and radiometric calibrations (Grossmann et al., 2018; Pacheco-Labrador et al., 2019). Since instruments for SIF need sufficient spectral resolution to resolve absorption features such as oxygen bands or Fraunhofer lines (see SIF retrievals section for more details), the wavelength calibration requirements are more exacting than for hyperspectral instruments. Radiometric calibration of SIF instruments requires more care and attention than hyperspectral sensing methods because SIF is a measure of emitted energy, and errors in absolute irradiance do not cancel out as ratioed quantities, as is the case with reflectance (Balzarolo et al., 2011; Zhang et al., 2021). The response of sensors to light is often nonlinear, which can be particularly problematic for SIF accuracy because SIF retrievals rely on the depths of ‘dark’ absorption features (oxygen bands or Fraunhofer lines (Grossmann et al., 2018)). Existing studies report a wide range of techniques and frequencies for performing radiometric calibration (Meroni et al., 2009; Mohammed et al., 2019; Sun, Wen, et al., 2023), which may further contribute to discrepancies between SIF data across sites. The gold standard for radiometric calibrations is generally using a NIST-traceable integrating sphere (Butler et al., 2003), but this cannot easily be done in the field. We recommend that (1) studies report the specifics of their calibration strategy as well as examine changes in calibration over time, and (2) the broader SIF community develop a set of standard best practices for radiometric calibration. In general, radiometric calibrations should be performed a few times per year under a wide range of conditions, unless spectrometers are stored in a temperature and humidity-controlled box to reduce sensor drift (S1.1, (Grossmann et al., 2018)). A new calibration should also be performed whenever a component of the light path is changed or disconnected/reconnected, as modifications to the light path will impact the radiometric calibration. For SIF retrieval methods that rely on $O_2$ absorption features, an atmospheric correction to compensate for absorption of SIF by $O_2$ within the path from vegetation to sensor is also necessary (Sabater et al., 2018; van der Tol et al., 2023).

SIF faces the additional challenge of being a derived quantity that has no independent measurement standard for verification. More research and development could help develop an

independent standard for SIF measurements such as LEDs (Burkart et al., 2015) or fluorescent paint (Kneer et al., 2023). In the absence of a quantitative standard, users can measure the Kautsky effect (whereby fluorescence spikes and then rapidly decays as a response to rapid illumination, (Kautsky & Hirsch, 1931; Lichtenthaler, 1993) as a qualitative verification that an instrument is successfully detecting SIF (Burkart et al., 2015; Grossmann et al., 2018; Marrs et al., 2021; X. Yang et al., 2018). This verification is mostly only feasible in settings where a leaf or plant canopy can be quickly transitioned from complete darkness to light. This can be accomplished in a laboratory setting or in the field by covering or wrapping with reflective material (e.g. mylar insulation sheets) to dark-acclimate vegetation without inducing heat stress. Reporting of signal-to-noise ratios (SNR) of final SIF retrieval values, as well as of downwelling irradiance and reflected radiance measurements, can also be a useful tool to assess data reliability or filter data when SNR values fall below recommended thresholds (European Space Agency et al., 2015). A useful definition of SNR for SIF applications is the mean signal divided by one standard deviation of the fluctuations of the signal (Swayze et al., 2003).

***Metadata***

Detailed documentation of metadata and fully traceable data processing chains are necessary for improved multisite comparison of SIF data. As discussed above, the components of instrumentation used for measuring SIF may vary widely across sites, which can make published SIF values challenging to compare with one another (Marrs et al., 2021). Even seemingly trivial aspects such as type of cosine receptor can have a significant impact on instrument performance (Burkart et al., 2022; Gu, Wood, et al., 2019), thus metadata should include documentation of all instrument components so that users can better account for hardware-related uncertainties when analyzing SIF data from multiple sites. We provide sample metadata for reporting SIF in S2.

***Support data***

Detailed ancillary data can considerably aid the interpretation of SIF data. Observed top-of-canopy SIF is affected by both canopy structure and physiology, which can be better disentangled by concurrently measuring visible-NIR hyperspectral reflectance to derive estimates of the escape ratio (P. Yang, van der Tol, et al., 2020; Zeng et al., 2019), pigment concentrations (J. A. Gamon et al., 2016; Gitelson et al., 2005; Zeng et al., 2022), and other structural parameters. Furthermore, as light is the primary driver of SIF, characterizing the light environment by measuring PAR, fPAR, and diffuse PAR provides context for the retrieved SIF (Chen et al., 2020; Jiménez et al., 2018; Z. Pierrat et al., 2021; Y. Wu et al., 2022; K. Yang et al., 2018). Users may also consider reporting SIF normalized by the intensity of the retrieved light ($SIF_{relative}$) as a proxy for the yield of fluorescence which is closely linked to the yield of photosynthesis at longer time scales (Butterfield et al., 2023; Parazoo et al., 2020a; Z. Pierrat et al., 2021). The use of $SIF_{relative}$ also bypasses the need for a radiometric calibration, although it is still recommended to assess for sensor drift over time. Additionally, regular measurements of leaf-level active fluorescence provide additional insight into the physiological component of SIF (Chang et al., 2021; Helm et al., 2020; Martini et al., 2022) and can be used to collect parameters required to run SIF-driven mechanistic models of photosynthesis based on the light reactions (Gu, Han, et al., 2019; Han et al., 2022; Z. Liu et al., 2022; Sun, Gu, et al., 2023).

**Notes S1.3. Thermal Infrared Radiation Best Practices**

TIR measurements are related to surface temperature via the Stefan-Boltzmann law (Equation 3), however, TIR received at a sensor cannot be directly converted into an object's temperature. This is because it is the combined radiation of the emitting object, the energy radiated by the surroundings and reflected off the object of interest, and the additional energy emitted from gases in the atmosphere, as attenuated by the atmosphere between the target and the sensor:

$$M_{tot} = \tau\varepsilon_{obj}M_{obj} + \tau(1-\varepsilon_{obj})\varepsilon_{reflect}M_{reflect} + (1-\tau)M_{atm} \quad \text{(Equation S1)}$$

where $M_{tot}$ is the radiation received at the sensor, $\tau$ is the atmospheric transmittance, (a function of humidity and the distance between sensor and target), $\varepsilon_{obj}$ is the emissivity of the object of interest, $M_{obj}$ is the TIR radiation emitted by the object of interest, $\varepsilon_{reflect}$ is the emissivity of the surroundings, $M_{reflect}$ is the energy radiated by the surroundings and reflected off the object of interest, and $M_{atm}$ is the additional energy emitted from gases in the atmosphere (Aubrechet et al., 2016). Solving for $M_{obj}$ and applying the Stefan-Botzmann Law (or an instrument-specific variation of the Law) will yield the object's temperature.

Instrumentation for TIR measurements broadly falls into three categories: Infrared Thermometers (IRTs), four-channel radiometers, and fixed-thermal cameras (Table S2). IRTs are small and easily deployable and measure thermal radiation in a particular footprint, although they are often un- or infrequently calibrated. Four-channel radiometers are typically mounted on the towers themselves and measure hemispherical incoming and outgoing shortwave and longwave radiative fluxes. Fixed TIR cameras are also often tower-mounted and capture TIR images at regular intervals (Johnston et al., 2021).

***Instrument setup and temporal aggregation***

Several publications have provided overviews of implementing TIR sensors at flux sites. For IRTs, best practices can be found in the NEON Sensor Command, Control and Configuration (C3) Document: Biological Temperature (SanClements et al., 2022). For four-channel radiometers, best practices can be found in Doughty et al., 2023; Guo et al., 2023; Thakur et al., 2022, which use tower radiometers to estimate canopy temperature. For fixed thermal cameras suggested best practices and operational guidance are provided in C. Still et al., 2019.

Much like spectral reflectance and SIF data, TIR measurements can be made as frequently as several times per minute. To facilitate cross-comparison with flux data, and integration into existing flux networks, we again recommend using the same averaging technique as AmeriFlux and FLUXNET, which use 15 minutes on either side of the reported 30-minute timestamp (Pastorello et al., 2020).

***Calibrations and Data Processing***

Users have several options for calibration, correction, and use of TIR data. These options make variable levels of assumptions on the contributions of different radiative components described in Equation S1:

**(1) Mechanistic correction of TIR measurements:** To obtain target temperature mechanistically, radiation emitted by the target must be isolated. This goal can be achieved

using ancillary measurements present at flux tower sites (relative humidity, air temperature, incoming longwave radiation), estimates of distance between the sensor and the target (Faye et al., 2016), and target emissivity (Johnston et al., 2021; S. K. Meerdink et al., 2019; Rubio et al., 1997). This “mechanistic” method is the most accurate correction choice when there are numerous targets of interest at varying distances from the sensor with potentially variable emissivity. It accommodates changing environmental conditions, as the correction is calculated at each time point. However, it also requires extensive ancillary measurements, and relies on some assumptions and approximations: higher-order reflections are typically ignored (Aubrecht et al., 2016), reflected radiation is usually assumed to originate only from the sky (see (Johnston et al., 2022; Kim et al., 2018) for exceptions), and emissivity estimates are sensitive to target structure and, in the case of vegetation, ontogeny (Richardson et al., 2021).

**(2) Empirical correction of TIR measurements:** Empirical correction of TIR measurements may be necessary in instances where “raw” radiometric images are difficult to obtain and the data have already been subjected to a factory “correction” by proprietary software (Kim et al., 2018). Ancillary measurements to which target temperatures are sensitive (relative humidity, air temperature, incoming longwave radiation) are still required. Briefly, empirical linear relationships between temperatures based on default vs. varied parameters are established. Those relationships may then be applied to adjust default temperatures to relevant conditions (Kim et al., 2018; Simpson et al., 2022).

**(3) Analysis of uncorrected TIR measurements (factory default):** Analysis of uncorrected TIR measurements may be appropriate if relative, rather than absolute, temperature is of interest (Kim et al., 2016; Pau et al., 2018), or if the error from the uncorrected parameter choice is expected to be minimal. If relative temperature is measured across space, targets must be similar distances from the sensor and have similar emissivity. If relative temperature is measured through time, atmospheric conditions affecting signal attenuation (relative humidity, air temperature) must be relatively consistent.

Documentation on which processing approach is used will facilitate intercomparison of TIR data across sites. Furthermore, while not required, we suggest placing temperature-controlled blackbodies (or a body with a known emissivity and temperature) to be used as calibration/validation panels within the instrument or FOV. These enable systematic evaluation (and possible correction) of the TIR data at regular time intervals, further ensuring data quality.

**Notes S1.4. Microwave Best Practices**

Most microwave sensors retrieve signals in specific regions of the microwave spectrum, namely the Ku-band (1.7-2.5 cm), X-band (2.4-3.75 cm), C-band (3.75-7.5 cm), and L-band (15-30 cm) (Figure 1). Shorter microwave wavelengths (e.g. Ku-band) experience higher attenuation and best capture near surface water dynamics such as canopy water content. Longer microwave wavelengths (e.g., L-band) penetrate significantly deeper and can be used to estimate water content of soils and total aboveground biomass (de Roo et al., 2001; Moesinger et al., 2020).

The most common proximal microwave remote sensing instruments include microwave radiometers (Holtzman et al., 2021) and global positioning system (GPS) receivers (Humphrey & Frankenberg, 2023; Larson, 2016; Larson & Small, 2014; Small et al., 2010, 2014). Microwave radiometers can be installed at the top of towers and pointed down toward the vegetation to

measure microwave frequencies from the Ku- to L-band at high temporal frequency (e.g., 10-minute interval) and a spatial resolution proportional to the height of the instrument above the surface (Table S3). Radiometers measure both V-polarization and H-polarization brightness temperatures, which can be used like satellite measurements of surface brightness temperature to estimate both soil moisture and VOD (Holtzman et al., 2021). Alternatively, GPS receivers can be installed at tower sites to measure the L-band microwave signals transmitted from the global navigation satellite system (GNSS) satellites. There are over one hundred active GNSS satellites, which can be identified by the GPS receiver to determine which satellite is being tracked, enabling the calculation of its azimuth and elevation. Individual satellite tracks repeat after a period that depends on the GNSS constellation. GPS receivers measure both a direct signal from a given satellite and an indirect signal that has been reflected off the vegetation and soil surface (Larson, 2016). These two signals can be combined to reveal changes in both soil moisture and vegetation water content at near-continuous temporal resolution and at a spatial footprint proportional to the height of the microwave antenna above the surface (Larson, 2016; Larson & Small, 2014; Small et al., 2010, 2014).

***Instrument setup***

Only a few studies have used tower-mounted microwave instruments at flux tower sites . For microwave radiometers, (Holtzman et al., 2021) provides a guide for instrument setup and technical specifications for installation at a forest flux tower site. For tower-mounted GPS instruments, (Humphrey & Frankenberg, 2023) and (Larson & Small, 2014) provide setup instructions and technical specifications that can be applied to instrument installations at both forest and grassland/shrubland flux tower sites, respectively. For forest sites, a dual-GPS setup with one instrument above the canopy measuring the direct signal and one below measuring the transmitted signal provides the best measurement configuration (Figure 1, sensors A and B, (Humphrey & Frankenberg, 2023)). For low-stature vegetation sites (grass, shrub, crop), a single GPS instrument can be used to measure the direct and indirect microwave signal (Figure 1 sensor A, (Larson & Small, 2014)).

***Retrievals and Calibrations***

For microwave radiometers, (Holtzman et al., 2021) provide an approach for deriving VOD from V-polarized brightness temperatures measured by the tower-based radiometer using a single-channel algorithm (SCA) based on the zeroth-order “tau–omega” radiative transfer model (Long & Ulaby, 2014; Mo et al., 1982). Instrument calibrations should be carried out over the study period using an ambient black body as a warm target and sky measurement as a cold target (Rowlandson et al., 2018). Additional research is needed to compare multiple VOD retrieval algorithms and instrument calibration approaches towards a standardized approach that will enable across-site comparisons.

For GPS receivers, signal-to-noise ratios (SNR; in decibels) are measured by the receiver, which represents the magnitude of the received signal power from each GNSS satellite compared to the background noise (Bilich et al., 2007). For forest sites with one instrument above the canopy measuring the direct signal and one below measuring the transmitted signal (Figure 1, sensors A and B), the SNR differences between the two sensors mostly depend on the transmissivity of the canopy, so VOD can be estimated from decibel differences (Humphrey &

Frankenberg, 2023). For grassland and shrubland sites with a single instrument, the difference in SNR between the direct signal and the indirect signal that bounces off the surface near the tower can be used to infer transmittance. Additionally, (Larson & Small, 2014) present an alternative method for converting differences in SNR into a normalized microwave reflectance index (NMRI), which is closely related to VOD (M. O. Jones et al., 2014). Depending on tower setup, the SNR measurement can also include contributions from indirect ground multipath reflections which may contain information about, for instance, soil water content (Larson, 2016). Calibration of GPS receivers should follow EarthScope Plate Boundary Observatory (PBO) site standards (Roesler & Larson, 2018) and care should be taken in evaluating GPS receiver footprints to ensure a dominant contribution from vegetation when deriving vegetation proxies.

**Notes S1.5. LiDAR Best Practices**

Considerations and best practices for TLS scans are detailed in existing review articles (Wilkes et al., 2017). Therefore, here we make general recommendations for main considerations for TLS at flux towers and suggest that readers refer to the cited literature for more specific guidance. The choices of scanner, configuration, sample design, software, derived metrics, and the timing of the scans depend on the research goal. Like many other optical sensors, a balance between data quality, instrument cost, speed of data collection, and level of expertise needs consideration.

For aboveground biomass estimation using TLS, a 10 m grid is recommended in 1 ha plots (Wilkes et al., 2017), enabling co-registration of multiple scans into a single point cloud. When capturing finer spatial detail such as leaf angle variations, a finer grid is recommended (Béland & Kobayashi, 2021). The scanning grid also depends on canopy status, with less occlusion in leaf-off periods of woody elements allowing coarser grid resolutions. Irregular scanning positions that maximize coverage and minimize occlusion may also be appropriate for targeting single trees or small areas. On-ground scans can be supplemented with scans from elevated viewpoints such as scaffolding or tower platforms if available. More recently, TLS data is being supplemented and merged with high-resolution drone LiDAR to minimize occlusion at the top of canopy (Terryn et al., 2022).

As for instrument choice, scanners differ significantly in price and data quality (Calders et al., 2020), which affects the resolution of the point clouds, SNR, and subsequent quality of the data outputs. Instruments with higher resolution and less noise (e.g., Riegl GmbH (Austria), Faro (USA), and Leica Geosystems (Switzerland)) are generally more expensive ($30,000-$150,000 USD), whereas lower cost LiDAR instruments (e.g., Ouster Inc. (USA), $3,000-$5,000 USD) may be sufficient for certain applications such as biodiversity monitoring and general biomass estimation (e.g., (Eitel et al., 2016; Proudman et al., 2022). A further consideration is whether the scanner will be stationary or mobile (e.g. Zeb Go, (GeoSLAM, Australia)), which impacts the speed of data capture and noise level.

TLS data is often processed with a combination commercial software and open-source algorithms, such as CloudCompare and RayCloudTools (Lowe & Stepanas, 2021). The community has been actively developing algorithms for tree segmentation, canopy structural trait calculation, and QSM computation (Burt et al., 2019; Stovall et al., 2021; Terryn et al., 2022). LiDAR data is typically stored as the LAS file format, an industry-standard extensible

binary format. Other formats include the ASCII PTX format and the more recent ray cloud format stored in PLY (Lowe & Stepanas, 2021). The latter contain information on free space (air) as well as surface geometry, further increasing functionality over point clouds alone.

The Global Terrestrial Laser Scanning (GTLS) Database (global-tls.net) represents a major recent effort in consolidating TLS data, pushing for standard best practices for TLS studies, and developing a global open-source tree-level 3D trait database. The lessons learned from the initial development of the GTLS database are reflective of the challenges common in consolidating like and complimentary proximal remote sensing instruments at a global scale. Generally, the most substantial initial hurdles to database consolidation are cataloging the location and availability of all previous TLS studies, followed by developing a consistent metadata format/scheme to describe the datasets. In the case of TLS, like other instruments amid ongoing research and development, a standardized data collection protocol is not yet agreed upon despite the suggestions of best practices (Wilkes et al., 2017). This between study variability creates difficulty for standardized metadata development. Studies that have developed higher-level LiDAR products (e.g. QSMs, leaf angle distributions, plant area volume density profiles, etc.) often do so using a diverse range of ever-changing techniques and algorithms, presenting a major issue upon global-scale consolidation and larger-scale studies of plant structure. However, promising validation efforts are continually being published demonstrating improvements in the speed and accuracy of the structural metrics and processing speeds.

The present solution implemented and suggested by the GTLS database is a standardized open-source processing pipeline/protocol that can be applied by individual research groups, reducing the potential bias in processing output due to algorithm selection. As such, the general suggestion for developing consistent data products in a global database of TLS data requires the application of these processing pipelines to raw TLS datasets. Despite data consistency challenges for the ongoing developing the GTLS database this effort presents a major opportunity for fostering international collaboration, enabling global studies of single tree architecture, improving Earth observation calibration and validation, and generally informing the role of plant structure in the function of the Earth system.

**Notes S2. Metadata recommendations for tower-mounted hyperspectral and SIF instruments**

The main objective is the definition of metadata information for proximal sensing hyperspectral and solar-induced fluorescence (SIF) systems needed from flux networks to improve the variable descriptions. The metadata information reported here should provide all the possible important info to interpret the variables (VIs, reflectance, SIF) coming from different field systems. It should be noted that the fields described here may not be applicable for all instrument types, therefore, some fields may be left blank. Additionally, these metadata do not include site information (such as lat/lon) as those would typically be included with standard flux metadata and do not need to be repeated here.

The metadata hereby defined will be used to define the BADM (Biological, Ancillary, Disturbance and Metadata), a protocol standardized across AmeriFlux, ICOS, Fluxnet and other networks.

The metadata contains information about:

1. Instrument
2. Set up
3. Variables

**Instrument**

| | | |
|---|---|---|
| Name | | |
| Serial Number | | |
| Responsible person | | |
| Spectrometer 1 | Spectral Range (nm) | |
| | Spectral Resolution (FWHM) | |
| | Spectral Sampling Interval | |
| | SNR (at full signal) | |
| | CCD cooling | Y/N |
| Spectrometer 2 | Spectral Range (nm) | |

| | | |
|---|---|---|
| | Spectral Resolution (FWHM) | |
| | Spectral Sampling Interval | |
| | SNR (at full signal) | |
| | CCD cooling | Y/N |
| Spectrometer 3 | Spectral Range (nm) | |
| | Spectral Resolution (FWHM) | |
| | Spectral Sampling Interval | |
| | SNR (at full signal) | |
| | CCD cooling | |
| FOV type | Bi conical<br>Bi hemispherical<br>Hemispherical conical | FOV degree |
| Dark Current Acquisition | Every cycle<br>Every n cycle | |
| Optimization | Every cycle<br>Every n cycle | 80% Dyn range |
| Nonlinearity | Applied on raw data<br>Applied in post processing | Type of correction (e.g., 7$^{th}$ order polynomial) |
| Cosine receptor type | Model: | Error at 80 degree SZA (e.g., Max 10%) |

| | | |
|---|---|---|
| Downwelling radiance measurement model | Sandwich (downwelling radiance is collected before and after upwelling) | |

**SET UP**

| | | | |
|---|---|---|---|
| Tower height | | | |
| Installation height | Downward channel | | |
| | Upward channel | | |
| Distance sensor - TOC | | | |
| Position on the tower | N-NE- E- SE- S-SW-W-NW | | |
| Azimuth degree | | | |
| Scan type | Fixed | Tilt (degree) | Pan (degree) |
| | View area | (m2) | |
| | Measured target | Single plant or mix | |
| | PTU | Tilt settings | Pan settings |
| | View area | Min Range (m2) | Max Range |
| | Measured target | Various plants | |
| Observation frequency | | | |
| Calibration date | | | |
| Calibration type | | | |

**VARIABLES – VIS***

| **Var name** | **Center banc** | **Bandwidth (FWHM)** | **Expression** | **Data value** | **Spectra** |
|---|---|---|---|---|---|
| NDVI | 800;670 | 10;10 | (a-b)/(a+b) | mean | R |
| PRI | 531;570 | 2;2 | (a-b)/(a+b) | mean | R |
| MTCI | 754;709;681 | 7;10;7 | (a-b)/(b+c) | mean | R |
| SR | 795;810 | 10;10 | a/b | mean | R |
| EVI | 800;670;480 | 10;10;10 | 2.5*(a-b)/(a+6*b-7.5*c+1) | mean | R |
| REP | 670;800;700;740 | 10;10;10;10 | 700+40*((a-b/2)-c)/(d-c) | mean | R |
| TCARI | 700;670;550;670 | 5;5;5;5 | 3*(a-b-0.2*(a-c)*a/d) | mean | R |
| REDCl | 785;725 | 15;5 | a/b-1 | mean | R |
| MCRI | 510;725;785 | 5;5;15 | c/(a-b) | mean | R |
| SPEC_RED_REFL | 650 | 80 | a | mean | R |
| SPEC_NIR_REFL | 850 | 80 | a | mean | R |
| L800 | 800 | 10 | a | mean | L |

*These variables represent some common VI’s but is not meant to be an exhaustive list. Furthermore, bandwidth values (FWHM) will vary depending on sensor type. The values used in

this table represent common ranges for calculation but do not represent steadfast rules for VI calculation.

**SIF**

| SIF variables | Retrieval Wavelength | Retrieval type |
|---|---|---|
| Red SIF | 687 | iFLD-SFM-SVD |
| Far red SIF | 760 | iFLD-SFM-SVD |
| Full spectrum | 650-800 | Spectfit |

## Notes S3. Existing Publicly Available Data

### Notes S3.1. Spectral Reflectance Data Availability

The PhenoCam Network (https://phenocam.nau.edu/webcam/) has a standardized data processing framework where RGB color channels are extracted and descriptive statistics (e.g., mean) for specific regions of interest (individual species or vegetation times) are calculated to estimate the onset and cessation of greenness (Richardson et al., 2018; Seyednasrollah et al., 2019). All software, (i.e., the PhenoCam R package (Hufkens et al., 2018)) and VI python and hardware specifications (Seyednasrollah et al., 2019) are openly available. The PhenoCam Network provides publicly accessible data from over 700 sites. The success and approach of the PhenoCam Network can serve as both a prime example of the utility of networked science (specifically tower-mounted remote sensing) and a resource for how to generate such a network (Richardson, 2023). The ICOS network plans to grow similar capabilities, matching protocols (Hufkens et al., 2018) and infrastructure to grow the number of core sites across Europe.

In addition to the PhenoCam Network, there are several non-standardized locations where researchers may access spectral reflectance data. The Spectral Network (SpecNet) has made considerable effort to identify individual flux sites with optical data (https://specnet.info/fieldsites/). However, SpecNet does not presently have data hosting capabilities and or curate specific products.

Additionally, there are a few networks which have spectral data for a variety of different ecosystems and vegetation types. These include the Ecological Spectral Information System (EcoSIS, https://ecosis.org/), SPECCHIO (https://specchio.ch/), the United States Geologic Survey (USGS) Spectral Library (https://www.usgs.gov/labs/spectroscopy-lab/science/spectral-library), and the Advanced Spaceborne Thermal Emission and Reflection (ASTER)/ECOsystem Spaceborne Thermal Radiometer Experiment on the Space Station (ECOSTRESS) Spectral Library (https://speclib.jpl.nasa.gov/). While these libraries are incredibly useful data repositories and references for spectral data, these data are not necessarily located at flux towers or processed in a way that facilitates intercomparison with flux data. These data tend to range in scale from the leaf to site level and can represent a variety of temporal resolutions that do not easily translate into timeseries information cross comparable with flux data. New initiatives could aim to foster greater synergies between spectral databases and flux networks.

### Notes S3.2. Solar-Induced Fluorescence Data Availability

Despite the current lack of a consensus surrounding data standards and processing, individuals can access SIF data through existing publications. These datasets are often quality controlled and rigorously checked and are considered reliable/robust datasets if care is taken to understand that differences across sites may be attributable to differences in instrumentation and processing. An overview of publicly available data at the time of this publication can be found in Table S4. While a more consolidated network with clear standards of practice and a centralized data hosting repository would considerably help facilitate intercomparison, a current overview of existing publicly available datasets can be found at https://climatesciences.jpl.nasa.gov/sif/download-data/tower/ and is open to contributions from future data producers.

**Notes S3.3. Thermal Infrared Radiation Data Availability**

We are aware of the following TIR datasets at flux tower sites; the dataset may be potentially incomplete due to the lack of coordinated metadata, underscoring the need for future collaborative efforts.

- **AmeriFlux**
  - At AmeriFlux sites, data collection methods can vary, depending on the Principal Investigator and site. However, most sites include longwave radiation measurements, which are essential for temperature calculations, mentioned above. Additionally, currently, 76 include a 'T_CANOPY' variable, typically derived from thermocouples, though it may occasionally originate from IRTs as indicated in the specific site metadata.
- **FLUXNET2015 Dataset**
  - For FLUXNET data, the calculation of temperature uses two standard variables (Pastorello et al., 2020):
    - LW_IN_F: Longwave radiation, incoming
    - LW_OUT: Longwave radiation, outgoing
- **Integrated Carbon Observation System (ICOS)**
  - The ICOS Ecosystem Thematic Centre (ETC) developed guidelines for measurement of radiation across the certified stations (Nicolini et al., 2017).
- **National Ecological Observatory Network (NEON)**
  - Biological temperature, identified by NEON product ID DP1.00005.001, is derived from IRT measurements in two temporal resolutions: one-minute and thirty-minute averages. These are positioned within the soil array and at multiple heights on the tower infrastructure. Data availability spans from December 2013 to August 2023, encompassing a total of 47 unique sites.
- **Fixed-thermal cameras at flux-tower sites**
  - In addition to these datasets with IRT measurements, there are many eddy covariance sites with data from fixed-thermal cameras. Many of these data are not yet publicly available, limiting their broader use. Future efforts could develop a network of fixed-thermal cameras modeled after the PhenoCam Network.

**Notes S3.4. Microwave Data Availability**

Microwave radiometers or GPS receivers have rarely been installed at eddy covariance sites and usually for brief periods (Holtzman et al., 2021). However, over 1,000 GPS base stations with nearly identical instrumentation (GPS receivers and antennas) have been in operation since 2007 across the western United States with a primary focus on measuring the deformation of the Pacific-North America plate boundary as part of the PBO initiative (earthscope.org). PBO sites near flux towers could be used for initial comparisons (M. O. Jones et al., 2014). These existing sites are limited to low vegetation biomass areas to minimize off nadir reflectance contamination of the GPS signal. Data from existing GPS towers can be directly downloaded from The Geodetic Facility for the Advancement of Geoscience (GAGE) data

archive center (unavco.org). Additionally, GPS receiver and antenna can be rented at very low costs in support of funded projects. The expansion of microwave radiometers or the less costly GPS receivers across eddy covariance flux tower sites could revolutionize our understanding of vegetation water content dynamics in space and time (Geremia-Nievinski & Hobiger, 2021; Humphrey & Frankenberg, 2023).

**Notes S3.5. LiDAR Data Availability**

Because TLS has more developed standards of practice than other proximal remote sensing types there have been several successful recent efforts to consolidate TLS datasets into publicly available databases (Table S5). The existence and value of these databases highlights the immense potential for networked remote sensing science.

**Table S1.** Instrument descriptions for spectral reflectance. All instruments can be tower-mounted and therefore their spatial resolution ranges from cm to m and temporal resolution ranges from a few seconds to a few minutes. Note that this is not an exhaustive list of all sensors on the market, but some of the most common.

| **Instrument type** | **Spectral Bandwidth** | **Recommended Calibrations** | **Example Instruments** | **Example Studies** |
|---|---|---|---|---|
| Multispectral RGB | ~20-100 nm | | • PhenoCam<br>• FROST<br>• ams-OSRAM AG AS7262, AS7263 | (Hufkens et al., 2018; Richardson et al., 2018; Tomelleri et al., 2022; Heusinkveld et al., 2023) |
| Multispectral reflectance | 10 nm | Sensor cross-calibration | • Spectral Reflectance Sensors, METER, Apogee<br>• NDVI GreenSeeker<br>• Skye Instruments SKI | (J. A. Gamon et al., 2015; Garrity et al., 2011; Gracia-Romero et al., 2019; Harris et al., 2014; Magney, Eitel, et al., 2016; Magney, Vierling, et al., 2016) |
| Hyperspectral visible, NIR and SWIR | 1-10 nm | Dark current, wavelength and radiometric | • ASDFieldSpec<br>• Spectra Vista Corporation HR-1024i<br>• Spectral Evolution RS-3500, 8800, PSR+, NaturaSpec | (Sakowska et al., 2014; Hamada et al., 2020; C. Y. Wong et al., 2023) |
| Hyperspectral visible and NIR | 1-3 nm | Dark current, wavelength and radiometric | • FLAME-Ocean Insight<br>• VNIR N-Series, Headwall Photonics<br>• Unispec-DC spectro-radiometer | (Grossmann et al., 2018; Hilker et al., 2007; C. Y. S. Wong et al., 2023; Woodgate et al., 2020) |
| Ultra-hyperspectral visible and NIR | 0.3-2 nm | Dark current and radiometric | • RoX (JB Hyperspectral Devices GmbH, Germany)<br>• FloX (JB Hyperspectral Devices GmbH, Germany) | (Julitta et al., 2017; Naethe et al., 2023; Wagner et al., 2018) |

**Table S2.** Instrument descriptions for thermal infrared radiation. All instruments can be tower-mounted and therefore their spatial resolution ranges from cm to m and temporal resolution ranges from a few seconds to a few minutes. Note that this is not an exhaustive list of all sensors on the market, but some of the most used.

| **Instrument type** | **Recommended Calibrations** | **Example Models** | **Example Studies** |
|---|---|---|---|
| Infrared Thermometer (IRT) | Factory default, mechanistic | • Apogee SI-111-SS | (SanClements et al., 2022) |
| Four-channel radiometer | Factory default, mechanistic | • Kipp and Zonen CNR4 Net Radiometer<br>• Apogee SN-500-SS Net Radiometer | (Doughty et al., 2023; Guo et al., 2023; Thakur et al., 2022) |
| Fixed thermal camera | Factory default, mechanistic, empirical | • FLIR A700f<br>• ICI FMX 700 P-Series | (Aubrecht et al., 2016; Johnston et al., 2022; Kim et al., 2016; Pau et al., 2018; C. J. Still et al., 2021; Woodgate et al., 2020) |

**Table S3.** Instrument descriptions for proximal microwave measurements. All instruments can be tower-mounted and therefore their spatial resolution ranges from cm to m and up to near-continuous temporal resolution. Note that this is not an exhaustive list of all sensors on the market, but some of the most used.

| **Instrument type** | **Recommended Calibrations** | **Example Models** | **Example Studies** |
|---|---|---|---|
| Microwave radiometer | Warm target: ambient black body<br>Cold target: sky measurements | • PR-1475 radiometer (L-Band) | (Holtzman et al., 2021) |
| GNSS receiver and antenna | Factory default, mechanistic | • Trimble NETR9<br>• Septentrio PolaRx5 | 1/17/25 8:38:00 AM |

**Table S4.** Overview of existing publicly available site-level SIF datasets sorted by latitude. As data continue to be published users can find a more current version of this document and contribute at this link: https://climatesciences.jpl.nasa.gov/sif/download-data/tower/

| Site | Lat | Lon | Vegetation | Example Studies | Data Link(s) |
|---|---|---|---|---|---|
| Svalbard Integrated Arctic Earth Observing System (SIOS) | 78.18 | 15.92 | High-Arctic tundra vegetation | (Tømmervik et al., 2023) | https://thredds.met.no/thredds/catalog/arcticdata/infranor/NINA-FLOX/raw/catalog.html |
| National Ecological Observation Network (NEON) Delta Junction, USA | 63.88 | -145.75 | Evergreen Needleleaf and Understory vegetation | (Nelson et al., 2022; Z. A. Pierrat et al., 2022, 2024) | https://zenodo.org/records/5806488 https://doi.org/10.5281/zenodo.7231157 https://zenodo.org/records/10048770 |
| Southern Old Black Spruce (AmeriFlux site ID Ca-obs) | 53.98 | -105.12 | Mixed Evergreen Needleleaf and Deciduous Needleleaf | (Nehemy et al., 2023; Z. Pierrat et al., 2021, 2022; Z. A. Pierrat et al., 2022, 2024) | https://doi.org/10.5281/zenodo.7231157 https://doi.org/10.5281/zenodo.7596931 https://zenodo.org/records/10048770 |
| University of Michigan Biological Station AmeriFlux site (US-UMB) | 45.56 | -84.71 | Temperate Deciduous Forest | (Butterfield et al., 2023) | https://doi.org/10.7302/sx8c-y281 |
| Coles Field, Iowa | 42.49 | -93.52 | Corn | (Chang et al., 2020; He et al., 2020; Magney, Frankenberg, et al., 2019) | https://data.caltech.edu/records/em9wn-ntq87 |
| Harvard Forest Long-Term Ecological Research (LTER) | 42.32 | -72.10 | Temperate deciduous forest | (Lu et al., 2018; H. Yang et al., 2017) | https://harvardforest1.fas.harvard.edu/exist/apps/datasets/showData.html?id=HF283 |
| Brooks Field, Iowa | 41.97 | -93.69 | Soybean | (Chang et al., 2020; He et al., 2020; Magney, Frankenberg, et al., 2019) | https://data.caltech.edu/records/btwvg-rwr30 |
| China HuaiLai (HL) | 40.35 | 115.79 | Cropland | (Du et al., 2019) | https://zenodo.org/record/7244183 |
| China Xiao Tangshan (XTS) | 40.18 | 116.44 | Cropland | (Du et al., 2019) | https://zenodo.org/record/7244183 |
| Energy Farm of the University of Illinois at Urbana-Champaign, USA | 40.07 | -88.21 | Soybean | (Miao et al., 2018) | https://doi.org/10.13012/B2IDB-1329706_V1 |
| Niwot Ridge Colorado (AmeriFlux site id US-NR1) | 40.03 | -105.55 | Evergreen Needleleaf | (Magney, Bowling, et al., 2019; Parazoo et al., 2020b; Z. A. Pierrat et al., 2024; Raczka et al., 2019; J. C. Yang et al., 2022) | https://data.caltech.edu/records/meh5c-wy279 |

| | | | | | |
|---|---|---|---|---|---|
| Majadas de Tiétar, Cáceres, Extremadura, (FLUXNET site ES-LMa) | 39.56 | -5.45 | Mediterranean open woodland | (Martini et al., 2022) | https://zenodo.org/record/5773208 https://figshare.com/articles/dataset/Time_series_of_fluxes_biochemical_and_spectral_variables_simulated_with_SCOPE_model_for_Singular_Spectrum_Analysis/13190858 |
| National Institute of Standards and Technology, Gaithersburg, Maryland, USA | 39.13 | -77.22 | Temperate deciduous forest | (Marrs et al., 2020) | https://dataverse.harvard.edu/dataset.xhtml?persistentId=doi:10.7910/DVN/1GKVM4 |
| US Department of Agriculture's Agricultural Research Service (USDA-ARS) in Beltsville, MD, USA | 39.03 | -76.85 | Corn | (P. Yang, Van der Tol, et al., 2020) | https://doi.org/10.5194/bg-18-441-2021 (supplement) |
| China DaMan (DM) | 38.86 | 100.37 | Cropland | (Du et al., 2019) | https://zenodo.org/record/7244183 |
| China ARou (AR) | 38.04 | 100.46 | Grassland | (Du et al., 2019) | https://zenodo.org/record/7244183 |
| National Ecological Observation Network (NEON) Ordway-Swisher Biological Station | 29.67 | -81.99 | Evergreen Needleleaf | (Z. A. Pierrat et al., 2024) | https://zenodo.org/records/10048770 |

**Table S5.** Selection of available TLS datasets

| Data Description | Link | Notes |
|---|---|---|
| Global TLS database | https://www.global-tls.net/ | TLS data of forests collected by PIs from various institutions with >1000 plots including TERN TLS sites |
| TERN TLS | https://portal.tern.org.au/ | TLS scans collected across TERN sites |
| TLS scans in central Germany (12 plots) | https://doi.pangaea.de/10.1594/PANGAEA.942856 | The datasets include point clouds from a TLS scanner |
| UNAVCO | https://tls.unavco.org/projects/ | Several projects listed with TLS data |

## References


Alonso, L., Gomez-Chova, L., Vila-Frances, J., Amoros-Lopez, J., Guanter, L., Calpe, J., & Moreno, J. (2008). Improved Fraunhofer Line Discrimination Method for Vegetation Fluorescence Quantification. *IEEE Geoscience and Remote Sensing Letters*, *5*(4), 620–624. https://doi.org/10.1109/LGRS.2008.2001180

Aubrecht, D. M., Helliker, B. R., Goulden, M. L., Roberts, D. A., Still, C. J., & Richardson, A. D. (2016). Continuous, long-term, high-frequency thermal imaging of vegetation: Uncertainties and recommended best practices. *Agricultural and Forest Meteorology*, *228–229*, 315–326. https://doi.org/10.1016/j.agrformet.2016.07.017

Bai, G., Ge, Y., Leavitt, B., Gamon, J. A., & Scoby, D. (2023). Goniometer in the air: Enabling BRDF measurement of crop canopies using a cable-suspended plant phenotyping platform. *Biosystems Engineering*, *230*, 344–360. https://doi.org/10.1016/j.biosystemseng.2023.04.017

Balzarolo, M., Anderson, K., Nichol, C., Rossini, M., Vescovo, L., Arriga, N., Wohlfahrt, G., Calvet, J.-C., Carrara, A., Cerasoli, S., Cogliati, S., Daumard, F., Eklundh, L., Elbers, J. A., Evrendilek, F., Handcock, R. N., Kaduk, J., Klumpp, K., Longdoz, B., … Martín, M. P. (2011). Ground-Based Optical Measurements at European Flux Sites: A Review of Methods, Instruments and Current Controversies. *Sensors*, *11*(8), Article 8. https://doi.org/10.3390/s110807954

Béland, M., & Kobayashi, H. (2021). Mapping forest leaf area density from multiview terrestrial lidar. *Methods in Ecology and Evolution*, *12*(4), 619–633. https://doi.org/10.1111/2041-210X.13550

Bilich, A., Axelrad, P., & Larson, K. (2007, September 28). *Scientific Utility of the Signal-to-Noise Ratio (SNR) Reported by Geodetic GPS Receivers*. https://www.semanticscholar.org/paper/Scientific-Utility-of-the-Signal-to-Noise-Ratio-by-Bilich-Axelrad/1be665fbc355d0f63477e252c3b593141b247da5

Burkart, A., Kennedy, M., Näthe, P., & Julitta, T. (2022). Iterative design of a high light throughput cosine receptor fore optic for unattended proximal remote sensing. *Journal of Applied Remote Sensing*, *16*(4), 044513. https://doi.org/10.1117/1.JRS.16.044513

Burkart, A., Schickling, A., Mateo, M. P. C., Wrobel, T. J., Rossini, M., Cogliati, S., Julitta, T., & Rascher, U. (2015). A Method for Uncertainty Assessment of Passive Sun-Induced Chlorophyll Fluorescence Retrieval Using an Infrared Reference Light. *IEEE Sensors Journal*, *15*(8), 4603–4611. https://doi.org/10.1109/JSEN.2015.2422894

Burt, A., Disney, M., & Calders, K. (2019). Extracting individual trees from lidar point clouds using treeseg. *Methods in Ecology and Evolution*, *10*(3), 438–445. https://doi.org/10.1111/2041-210X.13121

Butler, J. J., Brown, S. W., Johnson, B. C., Biggar, S. F., Markham, B. L., Gracey, P. N., & Barnes, R. A. (2003). Radiometric Measurement Comparison on the Integrating Sphere Source Used to Calibrate the Moderate Resolution Imaging Spectroradiometer (MODIS) and the Landsat 7 Enhanced Thematic Mapper Plus (ETM+). *Journal of Research of the National Institute of Standards and Technology*, *108*(3).

Butterfield, Z., Magney, T., Grossmann, K., Bohrer, G., Vogel, C., Barr, S., & Keppel-Aleks, G. (2023). Accounting for Changes in Radiation Improves the Ability of SIF to Track Water Stress-Induced Losses in Summer GPP in a Temperate Deciduous Forest. *Journal of*

*Geophysical Research: Biogeosciences*, *128*(7), e2022JG007352. https://doi.org/10.1029/2022JG007352

Calders, K., Adams, J., Armston, J., Bartholomeus, H., Bauwens, S., Bentley, L. P., Chave, J., Danson, F. M., Demol, M., Disney, M., Gaulton, R., Krishna Moorthy, S. M., Levick, S. R., Saarinen, N., Schaaf, C., Stovall, A., Terryn, L., Wilkes, P., & Verbeeck, H. (2020). Terrestrial laser scanning in forest ecology: Expanding the horizon. *Remote Sensing of Environment*, *251*, 112102. https://doi.org/10.1016/j.rse.2020.112102

Campbell, P. K. E., Huemmrich, K. F., Middleton, E. M., Ward, L. A., Julitta, T., Daughtry, C. S. T., Burkart, A., Russ, A. L., & Kustas, W. P. (2019). Diurnal and Seasonal Variations in Chlorophyll Fluorescence Associated with Photosynthesis at Leaf and Canopy Scales. *Remote Sensing*, *11*(5), Article 5. https://doi.org/10.3390/rs11050488

Cendrero-Mateo, M. P., Wieneke, S., Damm, A., Alonso, L., Pinto, F., Moreno, J., Guanter, L., Celesti, M., Rossini, M., Sabater, N., Cogliati, S., Julitta, T., Rascher, U., Goulas, Y., Aasen, H., Pacheco-Labrador, J., Arthur, A. M., Cendrero-Mateo, M. P., Wieneke, S., … Arthur, A. M. (2019). Sun-Induced Chlorophyll Fluorescence III: Benchmarking Retrieval Methods and Sensor Characteristics for Proximal Sensing. *Remote Sensing 2019, Vol. 11, Page 962*, *11*(8), 962. https://doi.org/10.3390/RS11080962

Chang, C. Y., Guanter, L., Frankenberg, C., Köhler, P., Gu, L., Magney, T. S., Grossmann, K., & Sun, Y. (2020). Systematic Assessment of Retrieval Methods for Canopy Far-Red Solar-Induced Chlorophyll Fluorescence Using High-Frequency Automated Field Spectroscopy. *Journal of Geophysical Research: Biogeosciences*, *125*(7). https://doi.org/10.1029/2019JG005533/FORMAT/PDF

Chang, C. Y., Wen, J., Han, J., Kira, O., Levonne, J., Melkonian, J., Riha, S. J., Skovira, J., Ng, S., Gu, L., Wood, J. D., Näthe, P., & Sun, Y. (2021). Unpacking the drivers of diurnal dynamics of sun-induced chlorophyll fluorescence (SIF): Canopy structure, plant physiology, instrument configuration and retrieval methods. *Remote Sensing of Environment*, *265*(June), 112672. https://doi.org/10.1016/j.rse.2021.112672

Chen, J., Liu, X., Du, S., Ma, Y., & Liu, L. (2020). Integrating sif and clearness index to improve maize GPP estimation using continuous tower-based observations. *Sensors (Switzerland)*, *20*(9). https://doi.org/10.3390/s20092493

Chu, H., Luo, X., Ouyang, Z., Chan, W. S., Dengel, S., Biraud, S. C., Torn, M. S., Metzger, S., Kumar, J., Arain, M. A., Arkebauer, T. J., Baldocchi, D., Bernacchi, C., Billesbach, D., Black, T. A., Blanken, P. D., Bohrer, G., Bracho, R., Brown, S., … Zona, D. (2021). Representativeness of Eddy-Covariance flux footprints for areas surrounding AmeriFlux sites. *Agricultural and Forest Meteorology*, *301–302*, 108350. https://doi.org/10.1016/j.agrformet.2021.108350

Cogliati, S., Celesti, M., Cesana, I., Miglietta, F., Genesio, L., Julitta, T., Schuettemeyer, D., Drusch, M., Rascher, U., Jurado, P., & Colombo, R. (2019). A Spectral Fitting Algorithm to Retrieve the Fluorescence Spectrum from Canopy Radiance. *Remote Sensing*, *11*(16), Article 16. https://doi.org/10.3390/rs11161840

Cogliati, S., Rossini, M., Julitta, T., Meroni, M., Schickling, A., Burkart, A., Pinto, F., Rascher, U., & Colombo, R. (2015). Continuous and long-term measurements of reflectance and sun-induced chlorophyll fluorescence by using novel automated field spectroscopy systems.

*Remote Sensing of Environment*, *164*, 270–281. https://doi.org/10.1016/j.rse.2015.03.027

Daumard, F., Champagne, S., Fournier, A., Goulas, Y., Ounis, A., Hanocq, J.-F., & Moya, I. (2010). A Field Platform for Continuous Measurement of Canopy Fluorescence. *IEEE Transactions on Geoscience and Remote Sensing*, *48*(9), 3358–3368. https://doi.org/10.1109/TGRS.2010.2046420

de Roo, R. D., Du, Y., Ulaby, F. T., & Dobson, M. C. (2001). A semi-empirical backscattering model at L-band and C-band for a soybean canopy with soil moisture inversion. *IEEE Transactions on Geoscience and Remote Sensing*, *39*(4), 864–872. IEEE Transactions on Geoscience and Remote Sensing. https://doi.org/10.1109/36.917912

Doughty, C. E., Keany, J. M., Wiebe, B. C., Rey-Sanchez, C., Carter, K. R., Middleby, K. B., Cheesman, A. W., Goulden, M. L., Da Rocha, H. R., Miller, S. D., Malhi, Y., Fauset, S., Gloor, E., Slot, M., Oliveras Menor, I., Crous, K. Y., Goldsmith, G. R., & Fisher, J. B. (2023). Tropical forests are approaching critical temperature thresholds. *Nature*, *621*(7977), 105–111. https://doi.org/10.1038/s41586-023-06391-z

Du, S., Liu, L., Liu, X., Guo, J., Hu, J., Wang, S., & Zhang, Y. (2019). SIFSpec: Measuring Solar-Induced Chlorophyll Fluorescence Observations for Remote Sensing of Photosynthesis. *Sensors*, *19*(13), 3009. https://doi.org/10.3390/s19133009

Eitel, J. U. H., Höfle, B., Vierling, L. A., Abellán, A., Asner, G. P., Deems, J. S., Glennie, C. L., Joerg, P. C., LeWinter, A. L., Magney, T. S., Mandlburger, G., Morton, D. C., Müller, J., & Vierling, K. T. (2016). Beyond 3-D: The new spectrum of lidar applications for earth and ecological sciences. *Remote Sensing of Environment*, *186*, 372–392. https://doi.org/10.1016/j.rse.2016.08.018

European Space Agency, Fletcher, K., Rider, H., & European Space Agency (Eds.). (2015). *Report for mission selection*. ESA Communications.

Faye, E., Dangles, O., & Pincebourde, S. (2016). Distance makes the difference in thermography for ecological studies. *Journal of Thermal Biology*, *56*, 1–9. https://doi.org/10.1016/j.jtherbio.2015.11.011

Filippa, G., Cremonese, E., Migliavacca, M., Galvagno, M., Sonnentag, O., Humphreys, E., Hufkens, K., Ryu, Y., Verfaillie, J., Morra di Cella, U., & Richardson, A. D. (2018). NDVI derived from near-infrared-enabled digital cameras: Applicability across different plant functional types. *Agricultural and Forest Meteorology*, *249*, 275–285. https://doi.org/10.1016/j.agrformet.2017.11.003

Frankenberg, C., & Berry, J. (2017). Solar induced chlorophyll fluorescence: Origins, relation to photosynthesis and retrieval. *Comprehensive Remote Sensing*, *1–9*(1986), 143–162. https://doi.org/10.1016/B978-0-12-409548-9.10632-3

Gamon, J. A. (2015). Reviews and Syntheses: Optical sampling of the flux tower footprint. *Biogeosciences*, *12*(14), 4509–4523. https://doi.org/10.5194/bg-12-4509-2015

Gamon, J. A., Cheng, Y., Claudio, H., MacKinney, L., & Sims, D. A. (2006). A mobile tram system for systematic sampling of ecosystem optical properties. *Remote Sensing of Environment*, *103*(3), 246–254. https://doi.org/10.1016/j.rse.2006.04.006

Gamon, J. A., Coburn, C., Flanagan, L. B., Huemmrich, K. F., Kiddle, C., Sanchez-Azofeifa, G. A., Thayer, D. R., Vescovo, L., Gianelle, D., Sims, D. A., Rahman, A. F., & Pastorello, G. Z.

(2010). SpecNet revisited: Bridging flux and remote sensing communities. *Canadian Journal of Remote Sensing*, *36*(sup2), S376–S390. https://doi.org/10.5589/m10-067

Gamon, J. A., Huemmrich, K. F., Wong, C. Y. S., Ensminger, I., Garrity, S., Hollinger, D. Y., Noormets, A., & Peñuelask, J. (2016). A remotely sensed pigment index reveals photosynthetic phenology in evergreen conifers. *Proceedings of the National Academy of Sciences of the United States of America*, *113*(46), 13087–13092. https://doi.org/10.1073/pnas.1606162113

Gamon, J. A., Kovalchuck, O., Wong, C. Y. S., Harris, A., & Garrity, S. R. (2015). Monitoring seasonal and diurnal changes in photosynthetic pigments with automated PRI and NDVI sensors. *Biogeosciences*, *12*(13), 4149–4159. https://doi.org/10.5194/bg-12-4149-2015

Gamon, J., Rahman, a, Dungan, J., Schildhauer, M., & Huemmrich, K. (2006). Spectral Network (SpecNet)—What is it and why do we need it? *Remote Sensing of Environment*, *103*(3), 227–235. https://doi.org/10.1016/j.rse.2006.04.003

Garrity, S. R., Eitel, J. U. H., & Vierling, L. A. (2011). Disentangling the relationships between plant pigments and the photochemical reflectance index reveals a new approach for remote estimation of carotenoid content. *Remote Sensing of Environment*, *115*(2), 628–635. https://doi.org/10.1016/j.rse.2010.10.007

Geremia-Nievinski, F., & Hobiger, T. (2021). *Site guidelines for multi-purpose GNSS reflectometry stations*. https://doi.org/10.5281/zenodo.5335890

Gitelson, A. A., Viña, A., Ciganda, V., Rundquist, D. C., & Arkebauer, T. J. (2005). Remote estimation of canopy chlorophyll content in crops. *Geophysical Research Letters*, *32*(8). https://doi.org/10.1029/2005GL022688

Gracia-Romero, A., Kefauver, S. C., Fernandez-Gallego, J. A., Vergara-Díaz, O., Nieto-Taladriz, M. T., & Araus, J. L. (2019). UAV and Ground Image-Based Phenotyping: A Proof of Concept with Durum Wheat. *Remote Sensing*, *11*(10), Article 10. https://doi.org/10.3390/rs11101244

Grossmann, K., Frankenberg, C., Magney, T. S., Hurlock, S. C., Seibt, U., & Stutz, J. (2018). PhotoSpec: A new instrument to measure spatially distributed red and far-red Solar-Induced Chlorophyll Fluorescence. *Remote Sensing of Environment*, *216*(November 2017), 311–327. https://doi.org/10.1016/j.rse.2018.07.002

Gu, L., Han, J., Wood, J. D., Chang, C. Y. Y., & Sun, Y. (2019). Sun-induced Chl fluorescence and its importance for biophysical modeling of photosynthesis based on light reactions. *New Phytologist*, *223*(3), 1179–1191. https://doi.org/10.1111/nph.15796

Gu, L., Wood, J. D., Chang, C. Y. Y., Sun, Y., & Riggs, J. S. (2019). Advancing Terrestrial Ecosystem Science With a Novel Automated Measurement System for Sun-Induced Chlorophyll Fluorescence for Integration With Eddy Covariance Flux Networks. *Journal of Geophysical Research: Biogeosciences*, *124*(1), 127–146. https://doi.org/10.1029/2018JG004742

Guanter, L., Alonso, L., Gómez-Chova, L., Amorós-López, J., Vila, J., & Moreno, J. (2007). Estimation of solar-induced vegetation fluorescence from space measurements. *Geophysical Research Letters*, *34*(8). https://doi.org/10.1029/2007GL029289

Guanter, L., Rossini, M., Colombo, R., Meroni, M., Frankenberg, C., Lee, J.-E., & Joiner, J. (2013). Using field spectroscopy to assess the potential of statistical approaches for the retrieval

of sun-induced chlorophyll fluorescence from ground and space. *Remote Sensing of Environment*, *133*, 52–61. https://doi.org/10.1016/j.rse.2013.01.017

Guo, Z., Still, C. J., Lee, C. K. F., Ryu, Y., Blonder, B., Wang, J., Bonebrake, T. C., Hughes, A., Li, Y., Yeung, H. C. H., Zhang, K., Law, Y. K., Lin, Z., & Wu, J. (2023). Does plant ecosystem thermoregulation occur? An extratropical assessment at different spatial and temporal scales. *New Phytologist*, *238*(3), 1004–1018. https://doi.org/10.1111/nph.18632

Hamada, Y., Cook, D., & Bales, D. (2020). EcoSpec: Highly Equipped Tower-Based Hyperspectral and Thermal Infrared Automatic Remote Sensing System for Investigating Plant Responses to Environmental Changes. *Sensors*, *20*(19), Article 19. https://doi.org/10.3390/s20195463

Han, J., Chang, C. Y.-Y., Gu, L., Zhang, Y., Meeker, E. W., Magney, T. S., Walker, A. P., Wen, J., Kira, O., McNaull, S., & Sun, Y. (2022). The physiological basis for estimating photosynthesis from Chla fluorescence. *New Phytologist*, *234*(4), 1206–1219. https://doi.org/10.1111/nph.18045

Harris, A., Gamon, J. A., Pastorello, G. Z., & Wong, C. Y. S. (2014). Retrieval of the photochemical reflectance index for assessing xanthophyll cycle activity: A comparison of near-surface optical sensors. *Biogeosciences*, *11*(22), 6277–6292. https://doi.org/10.5194/bg-11-6277-2014

He, L., Magney, T., Dutta, D., Yin, Y., Köhler, P., Grossmann, K., Stutz, J., Dold, C., Hatfield, J., Guan, K., Peng, B., & Frankenberg, C. (2020). From the Ground to Space: Using Solar-Induced Chlorophyll Fluorescence to Estimate Crop Productivity. *Geophysical Research Letters*, *47*(7), e2020GL087474. https://doi.org/10.1029/2020GL087474

Helm, L. T., Shi, H., Lerdau, M. T., & Yang, X. (2020). Solar-induced chlorophyll fluorescence and short-term photosynthetic response to drought. *Ecological Applications*, *30*(5), e02101. https://doi.org/10.1002/eap.2101

Heusinkveld, B. G., Mol, W. B., & van Heerwaarden, C. C. (2023). A new accurate low-cost instrument for fast synchronized spatial measurements of light spectra. *Atmospheric Measurement Techniques*, *16*(15), 3767–3785. https://doi.org/10.5194/amt-16-3767-2023

Hilker, T., Coops, N. C., Nesic, Z., Wulder, M. A., & Black, A. T. (2007). Instrumentation and approach for unattended year round tower based measurements of spectral reflectance. *Computers and Electronics in Agriculture*, *56*(1), 72–84. https://doi.org/10.1016/j.compag.2007.01.003

Holtzman, N. M., Anderegg, L. D. L., Kraatz, S., Mavrovic, A., Sonnentag, O., Pappas, C., Cosh, M. H., Langlois, A., Lakhankar, T., Tesser, D., Steiner, N., Colliander, A., Roy, A., & Konings, A. G. (2021). L-band vegetation optical depth as an indicator of plant water potential in a temperate deciduous forest stand. *Biogeosciences*, *18*(2), 739–753. https://doi.org/10.5194/bg-18-739-2021

Hufkens, K., Basler, D., Milliman, T., Melaas, E. K., & Richardson, A. D. (2018). An integrated phenology modelling framework in r. *Methods in Ecology and Evolution*, *9*(5), 1276–1285. https://doi.org/10.1111/2041-210X.12970

Humphrey, V., & Frankenberg, C. (2023). Continuous ground monitoring of vegetation optical depth and water content with GPS signals. *Biogeosciences*, *20*(9), 1789–1811. https://doi.org/10.5194/bg-20-1789-2023

Jafarbiglu, H., & Pourreza, A. (2023). Impact of sun-view geometry on canopy spectral reflectance variability. *ISPRS Journal of Photogrammetry and Remote Sensing*, *196*, 270–286. https://doi.org/10.1016/j.isprsjprs.2022.12.002

Jiménez, M., Moncholí, A., Salido, E., & de Miguel, E. (2018). Testing the Measurability of Sun Induced Fluorescence Under Optimal and Non-Optimal Sky Conditions. *IGARSS 2018 - 2018 IEEE International Geoscience and Remote Sensing Symposium*, 3505–3508. https://doi.org/10.1109/IGARSS.2018.8518344

Johnston, M. R., Andreu, A., Verfaillie, J., Baldocchi, D., González-Dugo, M. P., & Moorcroft, P. R. (2021). Measuring surface temperatures in a woodland savanna: Opportunities and challenges of thermal imaging in an open-canopy ecosystem. *Agricultural and Forest Meteorology*, *310*, 108484. https://doi.org/10.1016/j.agrformet.2021.108484

Johnston, M. R., Andreu, A., Verfaillie, J., Baldocchi, D., & Moorcroft, P. R. (2022). What lies beneath: Vertical temperature heterogeneity in a Mediterranean woodland savanna. *Remote Sensing of Environment*, *274*, 112950. https://doi.org/10.1016/j.rse.2022.112950

Jones, M. O., Kimball, J. S., Small, E. E., & Larson, K. M. (2014). Comparing land surface phenology derived from satellite and GPS network microwave remote sensing. *International Journal of Biometeorology*, *58*(6), 1305–1315. https://doi.org/10.1007/s00484-013-0726-z

Jones, T. S., Logan, B. A., Reblin, J. S., Bombard, D. M., Ross, B. P., Allen, D. W., Marrs, J. K., & Hutyra, L. R. (2023). Stress-induced changes in photosynthesis and proximal fluorescence emission of turfgrass. *Environmental Research Communications*, *5*(11), 111005. https://doi.org/10.1088/2515-7620/ad0b29

Julitta, T., Burkart, A., Rossini, M., Schickling, A., Colombo, R., Rascher, U., & Cogliati, S. (2017). FLoX: A System for Automatic Long Term Measurements of Top of Canopy Sun Induced Chlorophyll Fluorescence. *FLEX 2017 Workshop, ESA-ESRIN. ESA: FLEX*. https://www.dropbox.com/s/w8umv5j9nvk5p5w/Abstract-book.pdf?dl=0

Julitta, T., Corp, L., Rossini, M., Burkart, A., Cogliati, S., Davies, N., Hom, M., Mac Arthur, A., Middleton, E., Rascher, U., Schickling, A., Colombo, R., Julitta, T., Corp, L. A., Rossini, M., Burkart, A., Cogliati, S., Davies, N., Hom, M., … Colombo, R. (2016). Comparison of Sun-Induced Chlorophyll Fluorescence Estimates Obtained from Four Portable Field Spectroradiometers. *Remote Sensing*, *8*(2), 122. https://doi.org/10.3390/rs8020122

Kautsky, H., & Hirsch, A. (1931). Neue Versuche zur Kohlensäureassimilation. *Naturwissenschaften*, *19*(48), 964–964. https://doi.org/10.1007/BF01516164

Kim, Y., Still, C. J., Hanson, C. V., Kwon, H., Greer, B. T., & Law, B. E. (2016). Canopy skin temperature variations in relation to climate, soil temperature, and carbon flux at a ponderosa pine forest in central Oregon. *Agricultural and Forest Meteorology*, *226–227*, 161–173. https://doi.org/10.1016/j.agrformet.2016.06.001

Kim, Y., Still, C. J., Roberts, D. A., & Goulden, M. L. (2018). Thermal infrared imaging of conifer leaf temperatures: Comparison to thermocouple measurements and assessment of environmental influences. *Agricultural and Forest Meteorology*, *248*, 361–371. https://doi.org/10.1016/j.agrformet.2017.10.010

Kneer, C., Burkart, A., Bongartz, J., Siegmann, B., Bendig, J., Jenal, A., & Rascher, U. (2023). A Snapshot Imaging System for the Measurement of Solar-Induced Chlorophyll

Fluorescence—Addressing the Challenges of High-Performance Spectral Imaging. *IEEE Sensors Journal*, *23*(19), 23255–23269. IEEE Sensors Journal. https://doi.org/10.1109/JSEN.2023.3297054

Larson, K. M. (2016). GPS interferometric reflectometry: Applications to surface soil moisture, snow depth, and vegetation water content in the western United States. *WIREs Water*, *3*(6), 775–787. https://doi.org/10.1002/wat2.1167

Larson, K. M., & Small, E. E. (2014). Normalized Microwave Reflection Index: A Vegetation Measurement Derived From GPS Networks. *IEEE Journal of Selected Topics in Applied Earth Observations and Remote Sensing*, *7*(5), 1501–1511. IEEE Journal of Selected Topics in Applied Earth Observations and Remote Sensing. https://doi.org/10.1109/JSTARS.2014.2300116

Lichtenthaler, H. K. (1993). The Kautsky effect: 60 years of chlorophyll fluorescence induction kinetics. *Photosynthetica 27 (1992) S. 45-55.*

Liu, X., Liu, L., Zhang, S., & Zhou, X. (2015). New Spectral Fitting Method for Full-Spectrum Solar-Induced Chlorophyll Fluorescence Retrieval Based on Principal Components Analysis. *Remote Sensing*, *7*(8), Article 8. https://doi.org/10.3390/rs70810626

Liu, Y., Wu, C., Sonnentag, O., Desai, A. R., & Wang, J. (2020). Using the red chromatic coordinate to characterize the phenology of forest canopy photosynthesis. *Agricultural and Forest Meteorology*, *285–286*, 107910. https://doi.org/10.1016/j.agrformet.2020.107910

Liu, Z., Zhao, F., Liu, X., Yu, Q., Wang, Y., Peng, X., Cai, H., & Lu, X. (2022). Direct estimation of photosynthetic CO2 assimilation from solar-induced chlorophyll fluorescence (SIF). *Remote Sensing of Environment*, *271*, 112893. https://doi.org/10.1016/j.rse.2022.112893

Long, D. & Ulaby. (2014). *F. Ulaby and D.G. Long, Microwave Radar and Radiometric Remote Sensing, University of Michigan Press, Ann Arbor, Michigan, 2014.*

Lowe, T. D., & Stepanas, K. (2021). RayCloudTools: A Concise Interface for Analysis and Manipulation of Ray Clouds. *IEEE Access*, *9*, 79712–79724. https://doi.org/10.1109/ACCESS.2021.3084954

Lu, X., Liu, Z., An, S., Miralles, D. G., Maes, W., Liu, Y., & Tang, J. (2018). Potential of solar-induced chlorophyll fluorescence to estimate transpiration in a temperate forest. *Agricultural and Forest Meteorology*, *252*, 75–87. https://doi.org/10.1016/j.agrformet.2018.01.017

Magney, T. S., Bowling, D. R., Logan, B. A., Grossmann, K., Stutz, J., Blanken, P. D., Burns, S. P., Cheng, R., Garcia, M. A., Köhler, P., Lopez, S., Parazoo, N. C., Raczka, B., Schimel, D., & Frankenberg, C. (2019). Mechanistic evidence for tracking the seasonality of photosynthesis with solar-induced fluorescence. *Proceedings of the National Academy of Sciences of the United States of America*, *116*(24), 11640–11645. https://doi.org/10.1073/pnas.1900278116

Magney, T. S., Eitel, J. U. H., Huggins, D. R., & Vierling, L. A. (2016). Proximal NDVI derived phenology improves in-season predictions of wheat quantity and quality. *Agricultural and Forest Meteorology*, *217*, 46–60. https://doi.org/10.1016/j.agrformet.2015.11.009

Magney, T. S., Frankenberg, C., Köhler, P., North, G., Davis, T. S., Dold, C., Dutta, D., Fisher, J. B., Grossmann, K., Harrington, A., Hatfield, J., Stutz, J., Sun, Y., & Porcar-Castell, A. (2019).

Disentangling Changes in the Spectral Shape of Chlorophyll Fluorescence: Implications for Remote Sensing of Photosynthesis. *Journal of Geophysical Research: Biogeosciences*, *124*(6), 1491–1507. https://doi.org/10.1029/2019JG005029

Magney, T. S., Vierling, L. A., Eitel, J. U. H., Huggins, D. R., & Garrity, S. R. (2016). Response of high frequency Photochemical Reflectance Index (PRI) measurements to environmental conditions in wheat. *Remote Sensing of Environment*, *173*, 84–97. https://doi.org/10.1016/j.rse.2015.11.013

Maier, S. W., Günther, K. P., & Stellmes, M. (2004). Sun-Induced Fluorescence: A New Tool for Precision Farming. In *Digital Imaging and Spectral Techniques: Applications to Precision Agriculture and Crop Physiology* (pp. 207–222). John Wiley & Sons, Ltd. https://doi.org/10.2134/asaspecpub66.c16

Marrs, J. K., Jones, T. S., Allen, D. W., & Hutyra, L. R. (2021). Instrumentation sensitivities for tower-based solar-induced fluorescence measurements. *Remote Sensing of Environment*, *259*, 112413. https://doi.org/10.1016/J.RSE.2021.112413

Marrs, J. K., Reblin, J. S., Logan, B. A., Allen, D. W., Reinmann, A. B., Bombard, D. M., Tabachnik, D., & Hutyra, L. R. (2020). Solar-induced fluorescence does not track photosynthetic carbon assimilation following induced stomatal closure. *Geophysical Research Letters*, 0–1. https://doi.org/10.1029/2020gl087956

Martini, D., Sakowska, K., Wohlfahrt, G., Pacheco-Labrador, J., van der Tol, C., Porcar-Castell, A., Magney, T. S., Carrara, A., Colombo, R., El-Madany, T. S., Gonzalez-Cascon, R., Martín, M. P., Julitta, T., Moreno, G., Rascher, U., Reichstein, M., Rossini, M., & Migliavacca, M. (2022). Heatwave breaks down the linearity between sun-induced fluorescence and gross primary production. *New Phytologist*, *233*(6), 2415–2428. https://doi.org/10.1111/nph.17920

Meerdink, S. K., Hook, S. J., Roberts, D. A., & Abbott, E. A. (2019). The ECOSTRESS spectral library version 1.0. *Remote Sensing of Environment*, *230*, 111196. https://doi.org/10.1016/j.rse.2019.05.015

Meerdink, S., Roberts, D., Hulley, G., Gader, P., Pisek, J., Adamson, K., King, J., & Hook, S. J. (2019). Plant species' spectral emissivity and temperature using the hyperspectral thermal emission spectrometer (HyTES) sensor. *Remote Sensing of Environment*, *224*, 421–435. https://doi.org/10.1016/j.rse.2019.02.009

Meroni, M., Barducci, A., Cogliati, S., Castagnoli, F., Rossini, M., Busetto, L., Migliavacca, M., Cremonese, E., Galvagno, M., Colombo, R., & Di Cella, U. M. (2011). The hyperspectral irradiometer, a new instrument for long-term and unattended field spectroscopy measurements. *Review of Scientific Instruments*, *82*(4), 043106. https://doi.org/10.1063/1.3574360

Meroni, M., Busetto, L., Colombo, R., Guanter, L., Moreno, J., & Verhoef, W. (2010). Performance of Spectral Fitting Methods for vegetation fluorescence quantification. *Remote Sensing of Environment*, *114*(2), 363–374. https://doi.org/10.1016/j.rse.2009.09.010

Meroni, M., Rossini, M., Guanter, L., Alonso, L., Rascher, U., Colombo, R., & Moreno, J. (2009). Remote sensing of solar-induced chlorophyll fluorescence: Review of methods and applications. *Remote Sensing of Environment*, *113*(10), 2037–2051. https://doi.org/10.1016/J.RSE.2009.05.003

Metzger, S., Junkermann, W., Mauder, M., Butterbach-Bahl, K., Trancón y Widemann, B., Neidl, F., Schäfer, K., Wieneke, S., Zheng, X. H., Schmid, H. P., & Foken, T. (2013). Spatially explicit regionalization of airborne flux measurements using environmental response functions. *Biogeosciences*, *10*(4), 2193–2217. https://doi.org/10.5194/bg-10-2193-2013

Miao, G., Guan, K., Yang, X., Bernacchi, C. J., Berry, J. A., DeLucia, E. H., Wu, J., Moore, C. E., Meacham, K., Cai, Y., Peng, B., Kimm, H., & Masters, M. D. (2018). Sun-Induced Chlorophyll Fluorescence, Photosynthesis, and Light Use Efficiency of a Soybean Field from Seasonally Continuous Measurements. *Journal of Geophysical Research: Biogeosciences*, *123*(2), 610–623. https://doi.org/10.1002/2017JG004180

Mo, T., Choudhury, B. J., Schmugge, T. J., Wang, J. R., & Jackson, T. J. (1982). A model for microwave emission from vegetation-covered fields. *Journal of Geophysical Research: Oceans*, *87*(C13), 11229–11237. https://doi.org/10.1029/JC087iC13p11229

Moesinger, L., Dorigo, W., de Jeu, R., van der Schalie, R., Scanlon, T., Teubner, I., & Forkel, M. (2020). The global long-term microwave Vegetation Optical Depth Climate Archive (VODCA). *Earth System Science Data*, *12*(1), 177–196. https://doi.org/10.5194/essd-12-177-2020

Mohammed, G. H., Colombo, R., Middleton, E. M., Rascher, U., van der Tol, C., Nedbal, L., Goulas, Y., Pérez-Priego, O., Damm, A., Meroni, M., Joiner, J., Cogliati, S., Verhoef, W., Malenovský, Z., Gastellu-Etchegorry, J. P., Miller, J. R., Guanter, L., Moreno, J., Moya, I., … Zarco-Tejada, P. J. (2019). Remote sensing of solar-induced chlorophyll fluorescence (SIF) in vegetation: 50 years of progress. *Remote Sensing of Environment*, *231*(February), 111177. https://doi.org/10.1016/j.rse.2019.04.030

Naethe, P., Asgari, M., Kneer, C., Knieps, M., Jenal, A., Weber, I., Moelter, T., Dzunic, F., Deffert, P., Rommel, E., Delaney, M., Baschek, B., Rock, G., Bongartz, J., & Burkart, A. (2023). Calibration and Validation from Ground to Airborne and Satellite Level: Joint Application of Time-Synchronous Field Spectroscopy, Drone, Aircraft and Sentinel-2 Imaging. *PFG – Journal of Photogrammetry, Remote Sensing and Geoinformation Science*, *91*(1), 43–58. https://doi.org/10.1007/s41064-022-00231-x

Naethe, P., Julitta, T., Chang, C. Y.-Y., Burkart, A., Migliavacca, M., Guanter, L., & Rascher, U. (2022). A precise method unaffected by atmospheric reabsorption for ground-based retrieval of red and far-red sun-induced chlorophyll fluorescence. *Agricultural and Forest Meteorology*, *325*, 109152. https://doi.org/10.1016/j.agrformet.2022.109152

Nehemy, M. F., Pierrat, Z., Maillet, J., Richardson, A. D., Stutz, J., Johnson, B., Helgason, W., Barr, A. G., Laroque, C. P., & McDonnell, J. J. (2023). Phenological assessment of transpiration: The stem-temp approach for determining start and end of season. *Agricultural and Forest Meteorology*, *331*, 109319. https://doi.org/10.1016/j.agrformet.2023.109319

Nelson, P. R., Maguire, A. J., Pierrat, Z., Orcutt, E. L., Yang, D., Serbin, S., Frost, G. V., Macander, M. J., Magney, T. S., Thompson, D. R., Wang, J. A., Oberbauer, S. F., Zesati, S. V., Davidson, S. J., Epstein, H. E., Unger, S., Campbell, P. K. E., Carmon, N., Velez-Reyes, M., & Huemmrich, K. F. (2022). Remote Sensing of Tundra Ecosystems Using High Spectral Resolution Reflectance: Opportunities and Challenges. *Journal of Geophysical Research: Biogeosciences*, *127*(2). https://doi.org/10.1029/2021JG006697

Nicolini, G., Sabbatini, S., & Papale, D. (2017). *ICOS Ecosystem Instructions for Radiation Measurements* [Portable Document Format (PDF)]. https://doi.org/10.18160/ZWGB-WRRX

Ocheltree, T. W., & Loescher, H. W. (2007). Design of the AmeriFlux Portable Eddy Covariance System and Uncertainty Analysis of Carbon Measurements. *Journal of Atmospheric and Oceanic Technology*, *24*(8), 1389–1406. https://doi.org/10.1175/JTECH2064.1

Pacheco-Labrador, Hueni, Mihai, Sakowska, Julitta, Kuusk, Sporea, Alonso, Burkart, Cendrero-Mateo, Aasen, Goulas, & Mac Arthur. (2019). Sun-Induced Chlorophyll Fluorescence I: Instrumental Considerations for Proximal Spectroradiometers. *Remote Sensing*, *11*(8), 960. https://doi.org/10.3390/rs11080960

Parazoo, N. C., Magney, T., Norton, A., Raczka, B., Bacour, C., Maignan, F., Baker, I., Zhang, Y., Qiu, B., Shi, M., MacBean, N., Bowling, D. R., Burns, S. P., Blanken, P. D., Stutz, J., Grossmann, K., & Frankenberg, C. (2020a). Wide discrepancies in the magnitude and direction of modeled solar-induced chlorophyll fluorescence in response to light conditions. *Biogeosciences*, *17*(13), 3733–3755. https://doi.org/10.5194/bg-17-3733-2020

Parazoo, N. C., Magney, T. S., Norton, A., Raczka, B., Bacour, C., Maignan, F., Baker, I., Zhang, Y., Qiu, B., Shi, M., MacBean, N., Bowling, D. R., Burns, S. P., Blanken, P. D., Stutz, J., Grossmann, K., & Frankenberg, C. (2020b). Wide discrepancies in the magnitude and direction of modelled SIF in response to light conditions. *Biogeosciences*, *February*, 1–42. https://doi.org/10.5194/bg-2019-508

Pastorello, G., Trotta, C., Canfora, E., Chu, H., Christianson, D., Cheah, Y.-W., Poindexter, C., Chen, J., Elbashandy, A., Humphrey, M., Isaac, P., Polidori, D., Reichstein, M., Ribeca, A., van Ingen, C., Vuichard, N., Zhang, L., Amiro, B., Ammann, C., … Papale, D. (2020). The FLUXNET2015 dataset and the ONEFlux processing pipeline for eddy covariance data. *Scientific Data*, *7*(1), Article 1. https://doi.org/10.1038/s41597-020-0534-3

Pau, S., Detto, M., Kim, Y., & Still, C. J. (2018). Tropical forest temperature thresholds for gross primary productivity. *Ecosphere*, *9*(7), e02311. https://doi.org/10.1002/ecs2.2311

Pierrat, Z. A., Bortnik, J., Johnson, B., Barr, A., Magney, T., Bowling, D. R., Parazoo, N., Frankenberg, C., Seibt, U., & Stutz, J. (2022). Forests for forests: Combining vegetation indices with solar-induced chlorophyll fluorescence in random forest models improves gross primary productivity prediction in the boreal forest. *Environmental Research Letters*, *17*(12), 125006. https://doi.org/10.1088/1748-9326/aca5a0

Pierrat, Z. A., Magney, T. S., Cheng, R., Maguire, A. J., Wong, C. Y. S., Nehemy, M. F., Rao, M., Nelson, S. E., Williams, A. F., Grosvenor, J. A. H., Smith, K. R., Reblin, J. S., Stutz, J., Richardson, A. D., Logan, B. A., & Bowling, D. R. (2024). The biological basis for using optical signals to track evergreen needleleaf photosynthesis. *BioScience*, biad116. https://doi.org/10.1093/biosci/biad116

Pierrat, Z., Magney, T., Parazoo, N. C., Grossmann, K., Bowling, D. R., Seibt, U., Johnson, B., Helgason, W., Barr, A., Bortnik, J., Norton, A., Maguire, A., Frankenberg, C., & Stutz, J. (2022). Diurnal and Seasonal Dynamics of Solar-Induced Chlorophyll Fluorescence, Vegetation Indices, and Gross Primary Productivity in the Boreal Forest. *Journal of Geophysical Research: Biogeosciences*, *127*(2), e2021JG006588. https://doi.org/10.1029/2021JG006588

Pierrat, Z., Nehemy, M. F., Roy, A., Magney, T., Parazoo, N. C., Laroque, C., Pappas, C., Sonnentag, O., Grossmann, K., Bowling, D. R., Seibt, U., Ramirez, A., Johnson, B., Helgason, W., Barr, A., & Stutz, J. (2021). Tower-Based Remote Sensing Reveals Mechanisms Behind a Two-phased Spring Transition in a Mixed-Species Boreal Forest. *Journal of Geophysical Research: Biogeosciences*, *126*(5), 1–20. https://doi.org/ve

Plascyk, J. A. (1975). The MK II Fraunhofer Line Discriminator (FLD-II) for Airborne and Orbital Remote Sensing of Solar-Stimulated Luminescence. *Optical Engineering*, *14*(4), 339–0. https://doi.org/10.1117/12.7971842

Porcar-Castell, A., Mac Arthur, A., Rossini, M., Eklundh, L., Pacheco-Labrador, J., Anderson, K., Balzarolo, M., Martín, M. P., Jin, H., Tomelleri, E., Cerasoli, S., Sakowska, K., Hueni, A., Julitta, T., Nichol, C. J., & Vescovo, L. (2015). EUROSPEC: At the interface between remote-sensing and ecosystem $CO_2$ flux measurements in Europe. *Biogeosciences*, *12*(20), 6103–6124. https://doi.org/10.5194/bg-12-6103-2015

Proudman, A., Ramezani, M., Digumarti, S. T., Chebrolu, N., & Fallon, M. (2022). Towards real-time forest inventory using handheld LiDAR. *Robotics and Autonomous Systems*, *157*, 104240. https://doi.org/10.1016/j.robot.2022.104240

Raczka, B., Porcar-Castell, A., Magney, T., Lee, J. E., Köhler, P., Frankenberg, C., Grossmann, K., Logan, B. A., Stutz, J., Blanken, P. D., Burns, S. P., Duarte, H., Yang, X., Lin, J. C., & Bowling, D. R. (2019). Sustained Nonphotochemical Quenching Shapes the Seasonal Pattern of Solar-Induced Fluorescence at a High-Elevation Evergreen Forest. *Journal of Geophysical Research: Biogeosciences*, *124*(7), 2005–2020. https://doi.org/10.1029/2018JG004883

Richardson, A. D. (2023). PhenoCam: An evolving, open-source tool to study the temporal and spatial variability of ecosystem-scale phenology. *Agricultural and Forest Meteorology*, *342*, 109751. https://doi.org/10.1016/j.agrformet.2023.109751

Richardson, A. D., Aubrecht, D. M., Basler, D., Hufkens, K., Muir, C. D., & Hanssen, L. (2021). Developmental changes in the reflectance spectra of temperate deciduous tree leaves and implications for thermal emissivity and leaf temperature. *New Phytologist*, *229*(2), 791–804. https://doi.org/10.1111/nph.16909

Richardson, A. D., Hufkens, K., Milliman, T., Aubrecht, D. M., Chen, M., Gray, J. M., Johnston, M. R., Keenan, T. F., Klosterman, S. T., Kosmala, M., Melaas, E. K., Friedl, M. A., & Frolking, S. (2018). Tracking vegetation phenology across diverse North American biomes using PhenoCam imagery. *Scientific Data*, *5*(1), Article 1. https://doi.org/10.1038/sdata.2018.28

Roesler, C., & Larson, K. M. (2018). Software tools for GNSS interferometric reflectometry (GNSS-IR). *GPS Solutions*, *22*(3), 80. https://doi.org/10.1007/s10291-018-0744-8

Rowlandson, T. L., Berg, A. A., Roy, A., Kim, E., Pardo Lara, R., Powers, J., Lewis, K., Houser, P., McDonald, K., Toose, P., Wu, A., De Marco, E., Derksen, C., Entin, J., Colliander, A., Xu, X., & Mavrovic, A. (2018). Capturing agricultural soil freeze/thaw state through remote sensing and ground observations: A soil freeze/thaw validation campaign. *Remote Sensing of Environment*, *211*, 59–70. https://doi.org/10.1016/j.rse.2018.04.003

Rubio, E., Caselles, V., & Badenas, C. (1997). Emissivity measurements of several soils and vegetation types in the 8–14, µm Wave band: Analysis of two field methods. *Remote*

*Sensing of Environment*, *59*(3), 490–521. https://doi.org/10.1016/S0034-4257(96)00123-X

Ryu, Y., Baldocchi, D. D., Verfaillie, J., Ma, S., Falk, M., Ruiz-Mercado, I., Hehn, T., & Sonnentag, O. (2010). Testing the performance of a novel spectral reflectance sensor, built with light emitting diodes (LEDs), to monitor ecosystem metabolism, structure and function. *Agricultural and Forest Meteorology*, *150*(12), 1597–1606. https://doi.org/10.1016/j.agrformet.2010.08.009

Sabater, N., Vicent, J., Alonso, L., Verrelst, J., Middleton, E. M., Porcar-Castell, A., & Moreno, J. (2018). Compensation of Oxygen Transmittance Effects for Proximal Sensing Retrieval of Canopy–Leaving Sun–Induced Chlorophyll Fluorescence. *Remote Sensing*, *10*(10), Article 10. https://doi.org/10.3390/rs10101551

SanClements, M., Loescher, H., & Thibault, K. (2022). *NEON SENSOR COMMAND, CONTROL AND CONFIGURATION (C3) DOCUMENT: BIOLOGICAL TEMPERATURE*.

Sakowska, K., Gianelle, D., Zaldei, A., MacArthur, A., Carotenuto, F., Miglietta, F., Zampedri, R., Cavagna, M., & Vescovo, L. (2015). WhiteRef: A New Tower-Based Hyperspectral System for Continuous Reflectance Measurements. *Sensors*, *15*(1), Article 1. https://doi.org/10.3390/s150101088

Scodellaro, R., Cesana, I., D'Alfonso, L., Bouzin, M., Collini, M., Chirico, G., Colombo, R., Miglietta, F., Celesti, M., Schuettemeyer, D., Cogliati, S., & Sironi, L. (2022). A novel hybrid machine learning phasor-based approach to retrieve a full set of solar-induced fluorescence metrics and biophysical parameters. *Remote Sensing of Environment*, *280*, 113196. https://doi.org/10.1016/j.rse.2022.113196

Seyednasrollah, B., Bowling, D. R., Cheng, R., Logan, B. A., Magney, T. S., Frankenberg, C., Yang, J. C., Young, A. M., Hufkens, K., Arain, M. A., Black, T. A., Blanken, P. D., Bracho, R., Jassal, R., Hollinger, D. Y., Law, B. E., Nesic, Z., & Richardson, A. D. (2020). Seasonal variation in the canopy color of temperate evergreen conifer forests. *New Phytologist*, nph.17046. https://doi.org/10.1111/nph.17046

Seyednasrollah, B., Young, A. M., Hufkens, K., Milliman, T., Friedl, M. A., Frolking, S., & Richardson, A. D. (2019). Tracking vegetation phenology across diverse biomes using Version 2.0 of the PhenoCam Dataset. *Scientific Data*, *6*(1), Article 1. https://doi.org/10.1038/s41597-019-0229-9

Simpson, J. E., Holman, F. H., Nieto, H., El-Madany, T. S., Migliavacca, M., Martin, M. P., Burchard-Levine, V., Cararra, A., Blöcher, S., Fiener, P., & Kaplan, J. O. (2022). UAS-based high resolution mapping of evapotranspiration in a Mediterranean tree-grass ecosystem. *Agricultural and Forest Meteorology*, *321*, 108981. https://doi.org/10.1016/j.agrformet.2022.108981

Small, E. E., Larson, K. M., & Braun, J. J. (2010). Sensing vegetation growth with reflected GPS signals. *Geophysical Research Letters*, *37*(12). https://doi.org/10.1029/2010GL042951

Small, E. E., Larson, K. M., & Smith, W. K. (2014). Normalized Microwave Reflection Index: Validation of Vegetation Water Content Estimates From Montana Grasslands. *IEEE Journal of Selected Topics in Applied Earth Observations and Remote Sensing*, *7*(5), 1512–1521. IEEE Journal of Selected Topics in Applied Earth Observations and Remote Sensing. https://doi.org/10.1109/JSTARS.2014.2320597

Still, C. J., Rastogi, B., Page, G. F. M., Griffith, D. M., Sibley, A., Schulze, M., Hawkins, L., Pau, S., Detto, M., & Helliker, B. R. (2021). Imaging canopy temperature: Shedding (thermal) light on ecosystem processes. *New Phytologist*, *230*(5), 1746–1753. https://doi.org/10.1111/nph.17321

Still, C., Powell, R., Aubrecht, D., Kim, Y., Helliker, B., Roberts, D., Richardson, A. D., & Goulden, M. (2019). Thermal imaging in plant and ecosystem ecology: Applications and challenges. *Ecosphere*, *10*(6), e02768. https://doi.org/10.1002/ecs2.2768

Stovall, A. E. L., Masters, B., Fatoyinbo, L., & Yang, X. (2021). TLSLeAF: Automatic leaf angle estimates from single-scan terrestrial laser scanning. *New Phytologist*, *232*(4), 1876–1892. https://doi.org/10.1111/nph.17548

Sun, Y., Gu, L., Wen, J., van der Tol, C., Porcar-Castell, A., Joiner, J., Chang, C. Y., Magney, T., Wang, L., Hu, L., Rascher, U., Zarco-Tejada, P., Barrett, C. B., Lai, J., Han, J., & Luo, Z. (2023). From remotely sensed solar-induced chlorophyll fluorescence to ecosystem structure, function, and service: Part I—Harnessing theory. *Global Change Biology*, *29*(11), 2926–2952. https://doi.org/10.1111/gcb.16634

Sun, Y., Wen, J., Gu, L., Joiner, J., Chang, C. Y., van der Tol, C., Porcar-Castell, A., Magney, T., Wang, L., Hu, L., Rascher, U., Zarco-Tejada, P., Barrett, C. B., Lai, J., Han, J., & Luo, Z. (2023). From remotely-sensed solar-induced chlorophyll fluorescence to ecosystem structure, function, and service: Part II—Harnessing data. *Global Change Biology*, *29*(11), 2893–2925. https://doi.org/10.1111/gcb.16646

Swayze, G. A., Clark, R. N., Goetz, A. F. H., Chrien, T. G., & Gorelick, N. S. (2003). Effects of spectrometer band pass, sampling, and signal-to-noise ratio on spectral identification using the Tetracorder algorithm. *Journal of Geophysical Research: Planets*, *108*(E9). https://doi.org/10.1029/2002JE001975

Terryn, L., Calders, K., Bartholomeus, H., Bartolo, R. E., Brede, B., D'hont, B., Disney, M., Herold, M., Lau, A., Shenkin, A., Whiteside, T. G., Wilkes, P., & Verbeeck, H. (2022). Quantifying tropical forest structure through terrestrial and UAV laser scanning fusion in Australian rainforests. *Remote Sensing of Environment*, *271*, 112912. https://doi.org/10.1016/j.rse.2022.112912

Thakur, G., Schymanski, S. J., Mallick, K., Trebs, I., & Sulis, M. (2022). Downwelling longwave radiation and sensible heat flux observations are critical for surface temperature and emissivity estimation from flux tower data. *Scientific Reports*, *12*(1), 8592. https://doi.org/10.1038/s41598-022-12304-3

Tomelleri, E., Belelli Marchesini, L., Yaroslavtsev, A., Asgharinia, S., & Valentini, R. (2022). Toward a Unified TreeTalker Data Curation Process. *Forests*, *13*(6), Article 6. https://doi.org/10.3390/f13060855

Tømmervik, H., Julitta, T., Nilsen, L., Park, T., Burkart, A., Ostapowicz, K., Karlsen, S. R., Parmentier, F.-J., Pirk, N., & Bjerke, J. W. (2023). The northernmost hyperspectral FLoX sensor dataset for monitoring of high-Arctic tundra vegetation phenology and Sun-Induced Fluorescence (SIF). *Data in Brief*, *50*, 109581. https://doi.org/10.1016/j.dib.2023.109581

Ustin, S. L., Roberts, D. A., Gamon, J. A., Asner, G. P., & Green, R. O. (2004). Using Imaging Spectroscopy to Study Ecosystem Processes and Properties. *BioScience*, *54*(6), 523–534. https://doi.org/10.1641/0006-3568(2004)054[0523:UISTSE]2.0.CO;2

van der Tol, C., Julitta, T., Yang, P., Sabater, N., Reiter, I., Tudoroiu, M., Schuettemeyer, D., & Drusch, M. (2023). Retrieval of chlorophyll fluorescence from a large distance using oxygen absorption bands. *Remote Sensing of Environment*, *284*, 113304. https://doi.org/10.1016/j.rse.2022.113304

Wagner, A., Hilgert, S., Kattenborn, T., & Fuchs, S. (2018). Proximal VIS-NIR spectrometry to retrieve substance concentrations in surface waters using partial least squares modelling. *Water Supply*, *19*(4), 1204–1211. https://doi.org/10.2166/ws.2018.177

Wang, N., Suomalainen, J., Bartholomeus, H., Kooistra, L., Masiliūnas, D., & Clevers, J. G. P. W. (2021). Diurnal variation of sun-induced chlorophyll fluorescence of agricultural crops observed from a point-based spectrometer on a UAV. *International Journal of Applied Earth Observation and Geoinformation*, *96*, 102276. https://doi.org/10.1016/j.jag.2020.102276

Wang, R., Gamon, J. A., Hmimina, G., Cogliati, S., Zygielbaum, A. I., Arkebauer, T. J., & Suyker, A. (2022). Harmonizing solar induced fluorescence across spatial scales, instruments, and extraction methods using proximal and airborne remote sensing: A multi-scale study in a soybean field. *Remote Sensing of Environment*, *281*, 113268. https://doi.org/10.1016/j.rse.2022.113268

Wilkes, P., Lau, A., Disney, M., Calders, K., Burt, A., Gonzalez de Tanago, J., Bartholomeus, H., Brede, B., & Herold, M. (2017). Data acquisition considerations for Terrestrial Laser Scanning of forest plots. *Remote Sensing of Environment*, *196*, 140–153. https://doi.org/10.1016/j.rse.2017.04.030

Wong, C. Y., Gilbert, M. E., Pierce, M. A., Parker, T. A., Palkovic, A., Gepts, P., Magney, T. S., & Buckley, T. N. (2023). Hyperspectral Remote Sensing for Phenotyping the Physiological Drought Response of Common and Tepary Bean. *Plant Phenomics*, *5*, 0021. https://doi.org/10.34133/plantphenomics.0021

Wong, C. Y. S. (2023). Plant optics: Underlying mechanisms in remotely sensed signals for phenotyping applications. *AoB PLANTS*, *15*(4), plad039. https://doi.org/10.1093/aobpla/plad039

Wong, C. Y. S., D'Odorico, P., Arain, M. A., & Ensminger, I. (2020). Tracking the phenology of photosynthesis using carotenoid-sensitive and near-infrared reflectance vegetation indices in a temperate evergreen and mixed deciduous forest. *New Phytologist*, *226*(6), 1682–1695. https://doi.org/10.1111/nph.16479

Wong, C. Y. S., Jones, T., McHugh, D. P., Gilbert, M. E., Gepts, P., Palkovic, A., Buckley, T. N., & Magney, T. S. (2023). TSWIFT: Tower Spectrometer on Wheels for Investigating Frequent Timeseries for high-throughput phenotyping of vegetation physiology. *Plant Methods*, *19*(1), 29. https://doi.org/10.1186/s13007-023-01001-5

Woodgate, W., Van Gorsel, E., Hughes, D., Suarez, L., Jimenez-Berni, J., & Held, A. (2020). THEMS: An automated thermal and hyperspectral proximal sensing system for canopy reflectance, radiance and temperature. *Plant Methods*, *16*(1), 105. https://doi.org/10.1186/s13007-020-00646-w

Wu, G., Guan, K., Jiang, C., Kimm, H., Miao, G., Yang, X., Bernacchi, C. J., Sun, X., Suyker, A. E., & Moore, C. E. (2023). Can upscaling ground nadir SIF to eddy covariance footprint improve the relationship between SIF and GPP in croplands? *Agricultural and Forest Meteorology*, *338*, 109532. https://doi.org/10.1016/j.agrformet.2023.109532

Wu, Y., Zhang, Z., Zhang, X., Wu, L., & Zhang, Y. (2022). How Do Sky Conditions Affect the Relationships Between Ground-Based Solar-Induced Chlorophyll Fluorescence and Gross Primary Productivity Across Different Plant Types? *Journal of Geophysical Research: Biogeosciences*, *127*(12), e2022JG006865. https://doi.org/10.1029/2022JG006865

Yang, H., Yang, X., Zhang, Y., Heskel, M. A., Lu, X., Munger, J. W., Sun, S., & Tang, J. (2017). Chlorophyll fluorescence tracks seasonal variations of photosynthesis from leaf to canopy in a temperate forest. *Global Change Biology*, *23*(7), 2874–2886. https://doi.org/10.1111/gcb.13590

Yang, J. C., Magney, T. S., Albert, L. P., Richardson, A. D., Frankenberg, C., Stutz, J., Grossmann, K., Burns, S. P., Seyednasrollah, B., Blanken, P. D., & Bowling, D. R. (2022). Gross primary production (GPP) and red solar induced fluorescence (SIF) respond differently to light and seasonal environmental conditions in a subalpine conifer forest. *Agricultural and Forest Meteorology*, *317*, 108904. https://doi.org/10.1016/J.AGRFORMET.2022.108904

Yang, J. C., Magney, T. S., Yan, D., Knowles, J. F., Smith, W. K., Scott, R. L., & Barron-Gafford, G. A. (2020). The Photochemical Reflectance Index (PRI) Captures the Ecohydrologic Sensitivity of a Semiarid Mixed Conifer Forest. *Journal of Geophysical Research: Biogeosciences*, *125*(11), e2019JG005624. https://doi.org/10.1029/2019JG005624

Yang, K., Ryu, Y., Dechant, B., Berry, J. A., Hwang, Y., Jiang, C., Kang, M., Kim, J., Kimm, H., Kornfeld, A., & Yang, X. (2018). *Sun-induced chlorophyll fluorescence is more strongly related to absorbed light than to photosynthesis at half-hourly resolution in a rice paddy*. Remote Sensing of Environment. https://doi.org/10.1016/j.rse.2018.07.008

Yang, P., van der Tol, C., Campbell, P. K. E., & Middleton, E. M. (2020). Fluorescence Correction Vegetation Index (FCVI): A physically based reflectance index to separate physiological and non-physiological information in far-red sun-induced chlorophyll fluorescence. *Remote Sensing of Environment*, *240*(January), 111676. https://doi.org/10.1016/j.rse.2020.111676

Yang, P., Van der Tol, C., Campbell, P., & Middleton, E. (2020). Unravelling the physical and physiological basis for the solar-induced chlorophyll fluorescence and photosynthesis relationship. *Biogeosciences Discussions*, 1–32. https://doi.org/10.5194/bg-2020-323

Yang, X., Shi, H., Stovall, A., Guan, K., Miao, G., Zhang, Y., Zhang, Y., Xiao, X., Ryu, Y., & Lee, J.-E. (2018). FluoSpec 2—An Automated Field Spectroscopy System to Monitor Canopy Solar-Induced Fluorescence. *Sensors*, *18*(7), Article 7. https://doi.org/10.3390/s18072063

Zeng, Y., Badgley, G., Dechant, B., Ryu, Y., Chen, M., & Berry, J. A. (2019). A practical approach for estimating the escape ratio of near-infrared solar-induced chlorophyll fluorescence. *Remote Sensing of Environment*, *232*(July), 111209. https://doi.org/won

Zeng, Y., Hao, D., Huete, A., Dechant, B., Berry, J., Chen, J. M., Joiner, J., Frankenberg, C., Bond-Lamberty, B., Ryu, Y., Xiao, J., Asrar, G. R., & Chen, M. (2022). Optical vegetation indices for monitoring terrestrial ecosystems globally. *Nature Reviews Earth & Environment*. https://doi.org/10.1038/s43017-022-00298-5

Zhang, Y., Zhang, Q., Liu, L., Zhang, Y., Wang, S., Ju, W., Zhou, G., Zhou, L., Tang, J., Zhu, X., Wang, F., Huang, Y., Zhang, Z., Qiu, B., Zhang, X., Wang, S., Huang, C., Tang, X., & Zhang, J. (2021). ChinaSpec: A Network for Long-Term Ground-Based Measurements of Solar-Induced Fluorescence in China. *Journal of Geophysical Research: Biogeosciences*, *126*(3), e2020JG006042. https://doi.org/10.1029/2020JG006042

Zhao, F., Guo, Y., Verhoef, W., Gu, X., Liu, L., & Yang, G. (2014). A Method to Reconstruct the Solar-Induced Canopy Fluorescence Spectrum from Hyperspectral Measurements. *Remote Sensing*, *6*(10), Article 10. https://doi.org/10.3390/rs61010171

Zhao, F., Li, R., Verhoef, W., Cogliati, S., Liu, X., Huang, Y., Guo, Y., & Huang, J. (2018). Reconstruction of the full spectrum of solar-induced chlorophyll fluorescence: Intercomparison study for a novel method. *Remote Sensing of Environment*, *219*, 233–246. https://doi.org/10.1016/j.rse.2018.10.021
